# LC-MS/MS metabolomics unravels the resistant phenotype of carbapenemase-producing Enterobacterales

**DOI:** 10.1007/s11306-025-02300-9

**Published:** 2025-08-12

**Authors:** Breanna Dixon, Waqar M. Ahmed, Stephen J. Fowler, Tim Felton, Drupad K. Trivedi

**Affiliations:** 1 Division of Immunology, Immunity to Infection and Respiratory Medicine, School of Biological Sciences, Faculty of Biology, Medicine and Health, University of Manchester, Manchester, UK; 2 Manchester Institute of Biotechnology, Department of Chemistry, University of Manchester, Manchester, UK; 3 Manchester University Hospitals NHS Foundation Trust, Manchester, UK

## Abstract

**Introduction:**

The degree of antimicrobial resistance demonstrated by carbapenemase-producing Enterobacterales (CPE) represents a growing public health challenge. Conventional methods for detecting CPE involve culture-based techniques with lengthy incubation steps. There is a need to develop rapid and accurate methods for the detection of resistance, for implementation into clinical diagnostics.

**Objectives:**

With cellular phenotype closely linked to the metabolome, the acquisition of resistance should result in detectable differences in microbial metabolism. Accordingly, we sought to profile the metabolome of Enterobacterales isolates belonging to both CPE and non-CPE groups to identify metabolites linked to CPE.

**Methods:**

We used liquid chromatography-mass spectrometry to profile the endo- and exometabolome of 32 *Klebsiella pneumoniae* and *Escherichia coli* isolates to identify metabolites which could predict CPE in antibiotic-free conditions after 6 h of growth.

**Results:**

Using supervised machine learning and multivariate analysis algorithms (partial least squares-discriminant analysis, *k*-nearest neighbour and random forest), we identified 21 metabolite biomarkers which displayed high performance metrics for the prediction of CPE (AUROCs ≥ 0.845). Results revealed a range of alterations between the metabolomes of CPE and non-CPE isolates. Pathway analysis revealed enrichment of microbial pathways including arginine metabolism, ATP-binding cassette transporters, purine metabolism, biotin metabolism, nucleotide metabolism, and biofilm formation, providing mechanistic insight into the resistance phenotype of CPE.

**Conclusion:**

Our models demonstrate the ability to distinguish CPE from non-CPE in under 7 h using metabolite biomarkers, showing potential for the development of a targeted diagnostic assay.

**Supplementary Information:**

The online version contains supplementary material available at 10.1007/s11306-025-02300-9.

## Introduction

Antimicrobial resistance is a growing global concern, in particular bacterial resistance to last-resort antibiotics such as carbapenems (Antimicrobial Resistance Collaborators, 2022). Carbapenems display broad-spectrum activity towards both Gram-positive and Gram-negative bacteria and are highly effective at treating multidrug-resistant bacteria such as Enterobacterales which produce extended spectrum beta-lactamases (ESBLs). However, increased incidence of carbapenem resistance in Enterobacterales has been observed globally in the last two decades (Brolund et al., 2019; Freeman et al., 2020; Kazmierczak et al., 2021; UK Health Security Agency, 2023). Three mechanisms of carbapenem resistance exist in Enterobacterales: enzyme production, efflux pumps and porin mutations. Enzymatic hydrolysis of antibiotics via carbapenemases is the main mechanism observed.

Conventional methods for detecting carbapenemase-producing Enterobacterales (CPE) involve culture-based techniques with lengthy incubation steps which can significantly delay the administration of appropriate treatment (Tamma & Simner, 2018). Curtailing the dissemination of CPE and ensuring the implementation of suitable and timely treatment regimens necessitates the development of new strategies for the detection of these organisms (World Health Organization, 2017). Whilst there have been advancements in matrix-assisted laser desorption ionization–time of flight mass spectrometry (MALDI-TOF MS) for the determination of antimicrobial susceptibility and resistance, these techniques are not without limitations (Lange et al., 2014). For example, despite shorter incubation times, the MALDI Biotyper antibiotic susceptibility test rapid assay (MBT-ASTRA) has a laborious protein extraction workflow and requires significant optimisation for each species and antibiotic (Idelevich & Becker, 2021). Furthermore, in assays which rely on the detection of antibiotic degradation peaks, carbapenemases with lower-hydrolytic activity such as OXA-48-like variants have been associated with reduced sensitivities (Johansson et al., 2014; Chong et al., 2015; Dortet et al., 2018).

CPE have been linked with higher mortality rates than other mechanisms of carbapenem resistance, suggesting the existence of additional interplaying factors aside from enzyme production (Borer et al., 2009; Tamma et al., 2017). Studies have demonstrated the contributions of accessory genes with otherwise unknown functions to AMR phenotype, and hence knowledge of the mechanisms underpinning the resistant phenotype remains incomplete (Yang & Wu, 2022).

Metabolites are involved at all stages of cellular processes. The presence or absence of specific metabolites may serve as a precise chemical signature of biological state at any given time. Modelling resistance on the basis of metabolomic signatures, therefore, may offer insight into the underlying molecular mechanisms associated with the resistant phenotype, as well as facilitate improved detection by elucidating potential biomarkers of resistance. Recent computational advances are unlocking the potential of metabolomics for characterising metabolites associated with particular phenotypes, enabling better identification of these metabolites (Johnson et al., 2016).

In this study, we sought to characterise the metabolome of *Klebsiella pneumoniae* and *Escherichia coli* isolates belonging to both CPE and non-CPE groups by analysing endometabolome and exometabolome using high resolution liquid chromatography–mass spectrometry (LC-MS). We aimed to uncover and identify discriminative metabolites with diagnostic potential for the prediction of CPE, in addition to metabolic pathways implicated in the underlying mechanisms of the resistant phenotype.

## Results

### Characterisation of bacterial isolates

To identify the acquired resistance genes present within the *Escherichia coli* and *Klebsiella pneumoniae* strains, we performed whole genome sequencing of 32 individual isolates. Analysis of WGS data indicated that 18 of 32 isolates were carbapenemase-producers. Five isolates harboured KPC genes (*bla*_KPC-2_ *n* = 4, *bla*_KPC-3_ *n* = 1), six harboured NDM genes (*bla*_NDM-1_ *n* = 3, *bla*_NDM-5_ *n* = 2, *bla*_NDM-7_ *n* = 1), and a further five harboured OXA-48-like genes (*bla*_OXA-48_ *n* = 3, *bla*_OXA-181_ *n* = 2) (Table 1; Supplementary Table 4). Two isolates contained both *bla*_NDM-1_ and *bla*_OXA-232_ genes concurrently whilst nine isolates were confirmed as ESBL-producing strains.

**Table 1:** Characteristics of bacterial isolates used in the study

				ANTIBIOTIC PROFILE					
ISOLATE	SPECIES	GROUP	SUBCLASS	MEM	IPM	CTX	AUG	MEM MIC (mg L^− 1^**)**	SOURCE
KP001	KP	NON-CPE	ESBL	S	S	R	R	0.064	GROIN SWAB
EC002	EC	NON-CPE	ESBL	S	S	R	R	0.032	CATHETER URINE
KP003	KP	NON-CPE	ESBL	S	S	R	R	0.032	BLOOD CULTURE
EC004	EC	NON-CPE	ESBL	S	S	R	R	0.064	BLOOD CULTURE
EC005	EC	NON-CPE	ESBL	S	S	R	R	0.032	BLOOD CULTURE
KP006	KP	CPE	KPC	R	R	R	R	128	BLOOD CULTURE
KP007	KP	CPE	KPC	R	R	R	R	> 128	BLOOD CULTURE
KP008	KP	CPE	KPC	R	R	R	R	32	UNKNOWN
KP009	KP	CPE	KPC	R	R	R	R	128	UNKNOWN
KP010	KP	CPE	KPC	R	R	R	R	32	URINE
EC011	EC	NON-CPE	ESBL	S	S	R	R	0.032	BLOOD CULTURE
KP012	KP	NON-CPE	ESBL	S	S	R	R	0.032	BLOOD CULTURE
EC013	EC	NON-CPE	NON	S	S	S	S	0.032	BLOOD CULTURE
KP014	KP	NON-CPE	ESBL	S	S	R	R	0.064	BLOOD CULTURE
EC015	EC	NON-CPE	ESBL	S	S	R	R	0.032	BLOOD CULTURE
EC016	EC	CPE	NDM	R	R	R	R	64	BLOOD CULTURE
KP017	KP	CPE	NDM	R	R	R	R	128	UNKNOWN
EC018	EC	CPE	OXA	I	R	R	R	0.25	BLOOD CULTURE
KP019	KP	CPE	OXA	I	R	I	R	2	UNKNOWN
EC020	EC	CPE	OXA	I	R	R	R	1	BLOOD CULTURE
EC021	EC	CPE	OXA	I	I	R	R	0.5	BLOOD CULTURE
KP022	KP	CPE	OXA-NDM	R	R	R	R	64	URINE
KP023	KP	CPE	OXA-NDM	R	R	R	R	128	BLOOD CULTURE
EC024	EC	NON-CPE	NON	S	S	S	S	0.032	UNKNOWN
KP025	KP	NON-CPE	NON	S	S	S	S	0.032	BRONCHIAL WASHING
EC026	EC	NON-CPE	NON	S	S	S	S	0.032	LABORATORY STRAIN
KP029	KP	NON-CPE	NON	S	S	S	S	0.125	UNKNOWN
KP030	KP	CPE	NDM	R	R	R	R	16	BRONCHIAL WASHING
KP031	KP	CPE	NDM	R	R	R	R	> 128	UNKNOWN
EC032	EC	CPE	NDM	R	R	R	R	128	RECTAL SWAB
EC033	EC	CPE	NDM	R	R	R	R	64	WOUND
KP034	KP	CPE	OXA	R	R	I	R	128	UNKNOWN

KP = *K. pneumoniae*, EC = *E. coli*, MEM = meropenem, IPM = imipenem, CTX = cefotaxime, AUG = Augmentin (amoxicillin-clavulanic acid), MIC = minimum inhibitory concentration, S = susceptible, I = susceptible (increased dose), R = resistant

Antibiotic susceptibility testing was performed using the Kirby-Bauer disk diffusion method. Bacterial isolates were screened against meropenem, imipenem, cefotaxime and amoxicillin-clavulanic acid and were denoted as resistant (R), susceptible (S), or susceptible – increased exposure (I) based on EUCAST guidelines for zone diameters (EUCAST, 2021). Susceptibility to both meropenem and imipenem in all isolates lacking a carbapenemase gene (*n* = 14) were revealed; five of which were susceptible to all tested antibiotics (Table 1). In total, 43.8% (*n* = 14) of all isolates showed resistance to meropenem, whilst 53.1% (*n* = 17) demonstrated resistance against imipenem. The susceptible – increased exposure phenotype (I) was seen only in the isolates which contained a single OXA-48-like gene as confirmed by WGS (*n* = 5), with 4/5 showing intermediate resistance to meropenem, 1/5 to imipenem and 2/5 to cefotaxime. The minimum inhibitory concentrations (MICs) of meropenem against the bacterial isolates were examined after inoculation in Mueller-Hinton broth for 18 h. Meropenem MIC values were mostly congruent with disk diffusion results, with the exception of four OXA-48-like isolates which exhibited MIC values in the susceptible range (≤ 2 mg L^− 1^) but displayed a susceptible – increased exposure phenotype with Kirby-Bauer.

### LC-MS metabolomic profiling of bacterial isolates

To characterise the metabolomic profile associated with CPE, we performed untargeted, high resolution liquid chromatography-mass spectrometry (LC-MS) analysis on the intracellular and extracellular extracts of individual bacterial isolates. To maximise our coverage of the metabolome, we employed hydrophilic interaction liquid chromatography (HILIC) and reversed-phase (RP)-LC operated in both positive (ESI+) and negative (ESI-) ion modes. After normalisation and filtering for data quality, 8,696 positive mode and 9,834 negative mode endometabolome features were detected in the HILIC data across all samples, whilst in RP 6,564 (ESI+) and 2,143 (ESI-) features were detected. For the exometabolome samples, quality filtering in addition to background subtraction yielded 340 (ESI+) and 460 (ESI-) features in HILIC, with a further 159 (ESI+) and 220 (ESI-) features in RP.

To assess initial separation between CPE and non-CPE based on all detected mass features, we performed the unsupervised multivariate analysis method namely principal component analysis (PCA) on each dataset (Supplementary Fig. 1). We did not observe strong clustering by CPE status. The most prominent clustering seen in the plots was associated with species (Supplementary Figs. 2 and 3). This analysis provided suitable rationale for the implementation of supervised machine learning methods for the purposes of data modelling and prediction of CPE.

### Application of supervised methods enables prediction of CPE

To identify metabolite markers that could discriminate between CPE and non-CPE bacteria, we performed feature selection on the intracellular and extracellular datasets. We split the data into training (70%) and test (30%) sets and selected three supervised machine learning algorithms [partial least squares-discriminant analysis (PLS-DA), *k*-nearest neighbours (kNN), and random forest (RF)]. We then tuned models on training set data with 5-fold cross validation repeated 10 times. To perform feature selection, we ranked features according to variable contributions towards each multivariate model for the comparison of CPE and non-CPE. The top 20% most discriminatory features for each model were selected, and models were reduced to those features which intersected between at least two of the three algorithms and which had a Bonferroni-corrected *p*-value of ≤ 0.05 (Supplementary Table 5).

### Predictive markers of CPE uncovered with tandem mass spectrometry

To obtain putative annotations for the discriminative mass features, we utilised LC-MS/MS with selective ion monitoring. Spectral library matching was performed against tandem MS data, enabling the putative assignment of 21 unique metabolites (12 endometabolome and 9 exometabolome) to Metabolomics Standards Initiative (MSI) level 2. With respect to the endometabolome samples, we were able to putatively annotate 10 unique metabolites detected using HILIC-LC-MS whilst two were annotated in the RP-LC-MS data (Table 2). We putatively identified four exometabolome metabolites from HILIC-MS and five metabolites from RP-LC-MS data (Table 2). We were able to further confirm 14 of these annotations to MSI level 1 by comparing the MS/MS spectra of the putatively annotated features with those of corresponding standard compounds (Supplementary Fig. 5–23). These were equally distributed between the endo- and exometabolome datasets.

**Table 2:** Discriminatory endometabolome metabolites for the prediction of CPE versus non-CPE. Only metabolites which could be putatively annotated are shown

	Putative Annotation	m/z	Adduct	Exact mass	MSI level
INTRACELLULAR					
HILIC ESI+	Pyridoxamine	169.6078	[M + H]+	168.0899	1
	Nicotinamide riboside	256.1013	[M + H]+	255.0981	2
	Biotin	267.0779	[M + Na]+	244.0882	1
	Uridine 5’-diphosphate (UDP)	405.0102	[M + H]+	404.0022	1
	Lipopolysaccharide (LPS) 14:2	466.2056	[M + H-H_2_O]+	465.2855	2
	UDP-glucose	605.0103	[M + K]+	566.0550	1
HILIC ESI-	Phenylpyruvate	163.0393	[M-H]-	164.0473	1
	Citrulline	174.1011	[M-H]-	175.0957	1
	Gluconic acid	194.0384	[M-H]-	196.0583	2
	3’,5’-cyclic adenosine monophosphate (cAMP)	328.0459	[M-H]-	329.0525	1
RP ESI+	Valine	299.1616	[2M + CH_3_CN + Na]+	117.0790	2
	Ceramide (Cer) 14:1;O2/10:0	466.3524	[M + NaHCOO + H]+	397.3556	2
EXTRACELLULAR					
HILIC ESI+	Hypoxanthine	159.0281	[M + Na]+	136.0385	1
	Arginine	174.0392	[M + H]+	174.1117	1
	Pantothenic acid	219.1062	[M + NH_4_-H_2_O]+	219.1107	1
	2’-Deoxyinosine	253.1009	[M + H]+	252.0859	1
RP ESI+	Choline	105.1110	[M + H]+	104.1075	1
	3-Hydroxytridecanoic acid	269.1388	[M + K]+	230.1882	2
RP ESI-	Ornithine	131.0704	[M-H]-	132.0899	1
	2’-Deoxycytidine	226.1085	[M-H]-	227.0906	1
	Cytidine diphosphate diacylglycerol (CDP-DG) (4:0/16:1(9Z))	260.0933	[M-3H]-3	783.3108	2

### Prediction of CPE yields high performance metrics

**Fig. 1:**
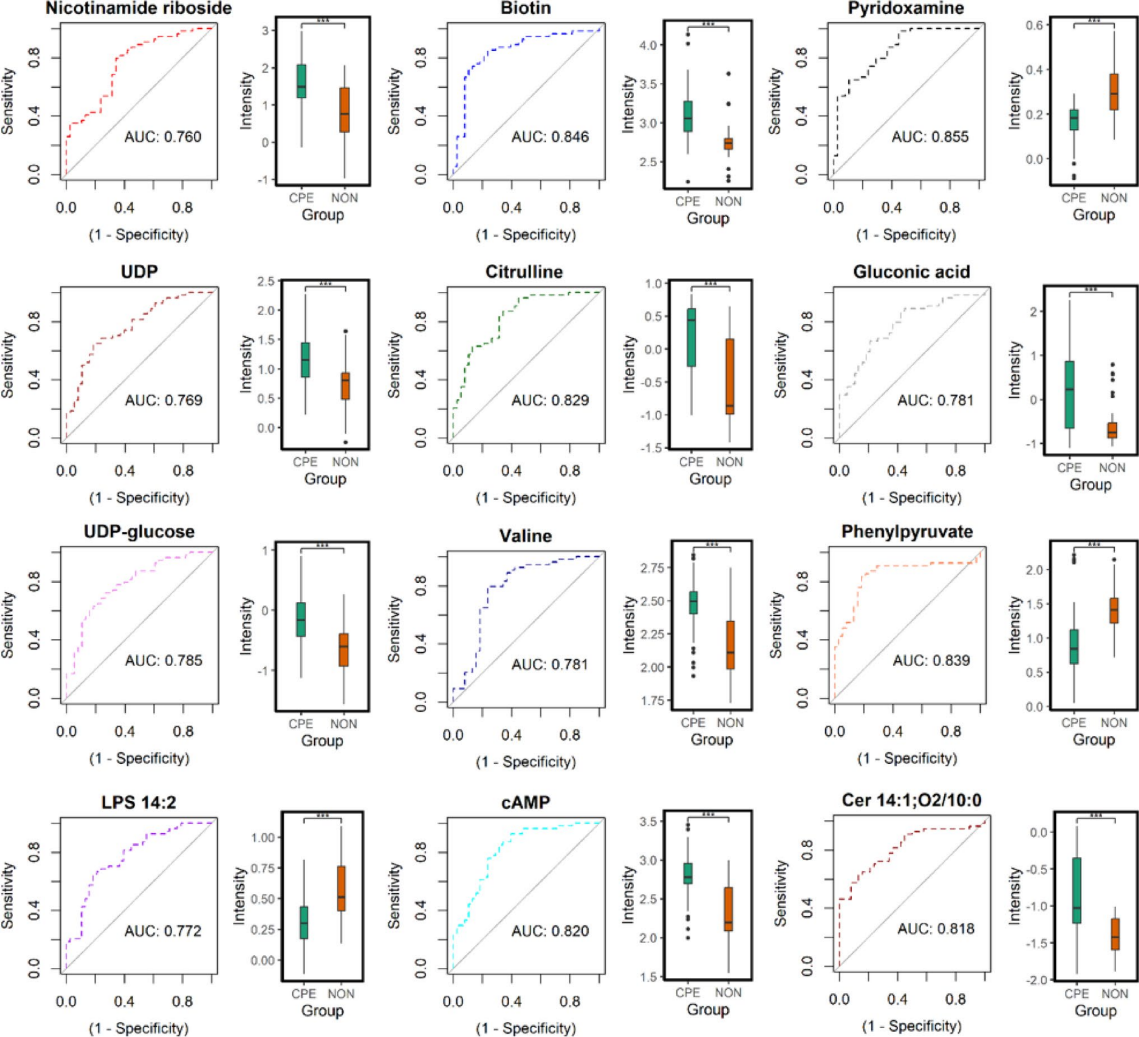
Individual ROC curves and boxplots for endometabolome metabolites annotated to MSI level 1 and 2. Outliers are denoted by black dots in the intensity box plots. Significance: *** *p ≤* 0.001

**Fig. 2:**
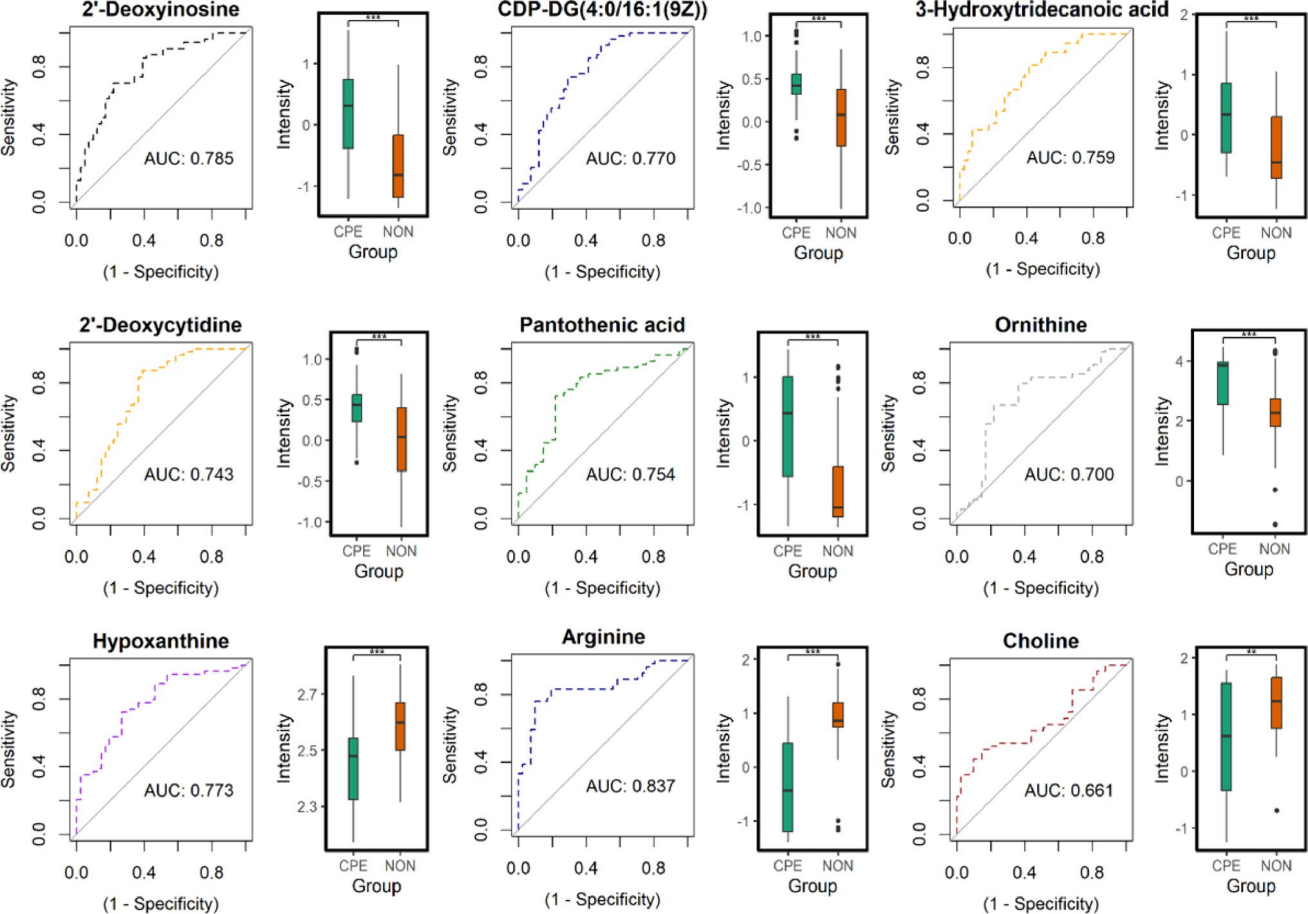
Individual ROC curves and boxplots for exometabolome metabolites annotated to MSI level 1 and 2. Outliers are denoted by black dots in the intensity box plots. Significance: *** *p ≤* 0.001, ** *p ≤* 0.01

To assess the predictive power of each individual metabolite for the prediction of CPE, we evaluated the receiver operating characteristics (ROCs) for each metabolite (Figs. 1 and 2). Amongst the endometabolome metabolites, the individual prediction of CPE revealed a minimum area under the ROC curve (AUC) of 0.760 (nicotinamide riboside). Six metabolites displayed AUCs greater than 0.800 (Cer 14:1;O2/10:0, AUC = 0.818; cAMP, AUC = 0.820; citrulline, AUC = 0.829; phenylpyruvate, AUC = 0.839; biotin, AUC = 0.846; and pyridoxamine, AUC = 0.855), indicating strong biomarker potential. Arginine (AUC = 0.837) was the only exometabolome metabolite to demonstrate an AUC greater than 0.800, with AUCs of the nine metabolites ranging 0.661–0.837.

**Fig. 3:**
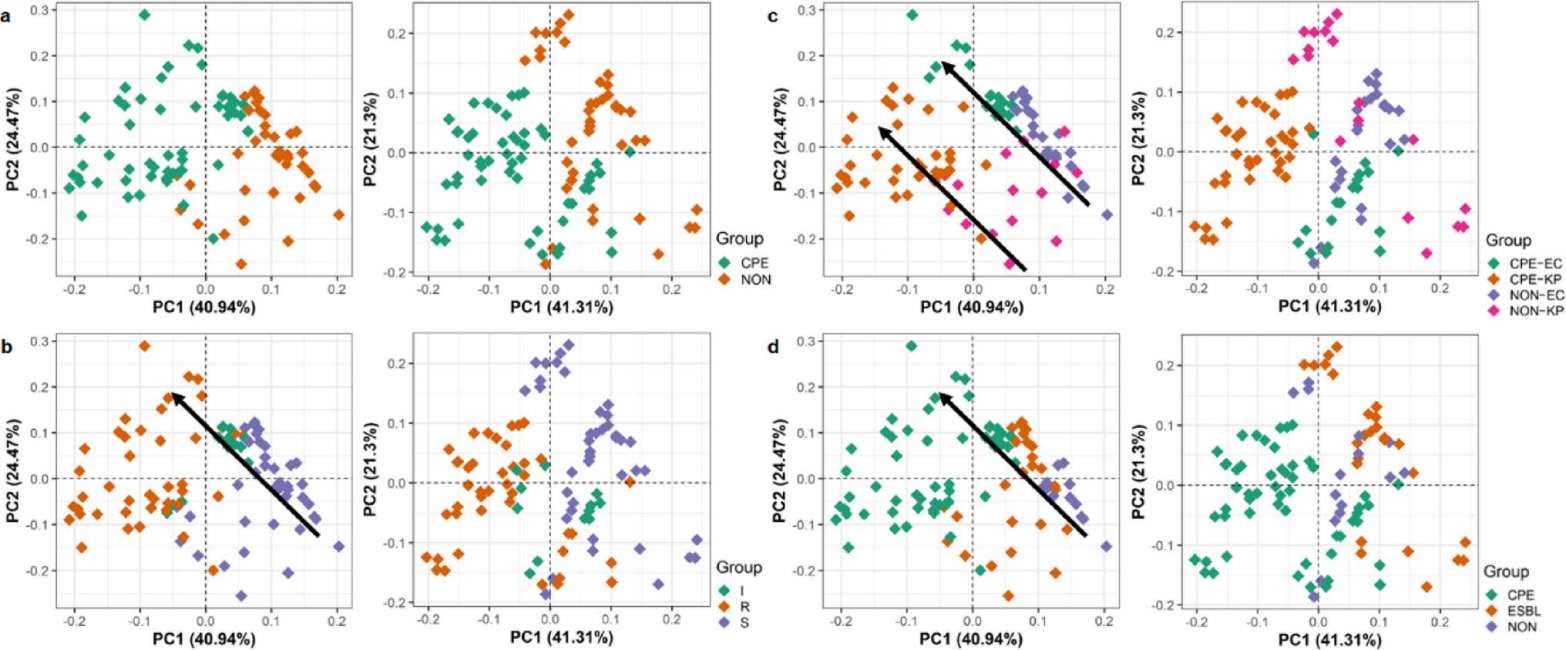
PCA scores plots based on the 12 annotated discriminative endometabolome and nine exometabolome metabolites from HILIC and RP analysis for the classification of CPE versus non-CPE. Class labels defined by CPE characterisation (CPE and non-CPE) (**A**), meropenem resistance phenotype (susceptible, susceptible – increased exposure, resistant) (**B**), species and genotype (**C**) and β-lactamase class (**D**). Left hand plots depict endometabolome data, whilst right hand plots depict exometabolome data. Arrows demonstrate directionality from least to most resistance

To visualise the discriminative ability of the identified metabolite markers, we produced PCA scores plots. PCA scores plots using all 12 putatively annotated discriminatory endometabolome metabolites demonstrated clear clustering of the CPE and non-CPE groups (Fig. 3A). PC1 explained 41% of the total variance, with CPE isolates appearing at low PC1 scores and separated from non-CPE isolates. Consistent with the endometabolome findings, CPE isolates were largely separated from non-CPE isolates along PC1 using the nine exometabolome discriminative metabolites, which explained 41% of the total variance (Fig. 3A).

To further investigate the observed trends in the PCA scores plots, we adjusted class labels to reflect susceptibility phenotype (meropenem S/I/R). The region of slight overlap that was seen in the original endometabolome binary genotype (CPE and non-CPE) scores plot (Fig. 3A) was revealed to be a cluster of isolates belonging to the susceptible – increased dose (I) phenotype (Fig. 3B). Further classification of the isolates based on genotype and species in combination revealed four distinct clusters across each quadrant of the scores plot (Fig. 3C). Dividing the non-CPE group into ESBL and non-producing bacteria also demonstrated that members of each group clustered amongst their own (Fig. 3D). Since ESBL-producers are also multidrug-resistant, although not to the same extent of carbapenemase-producers, we might expect these bacteria to display a phenotype in-between non-producers and CPE, which is demonstrated in the endometabolome plot. Similar patterns were seen in the exometabolome scores plots with alternative class labels, although the clustering was not as well defined as that observed in the endometabolome plots.

**Fig. 4:**
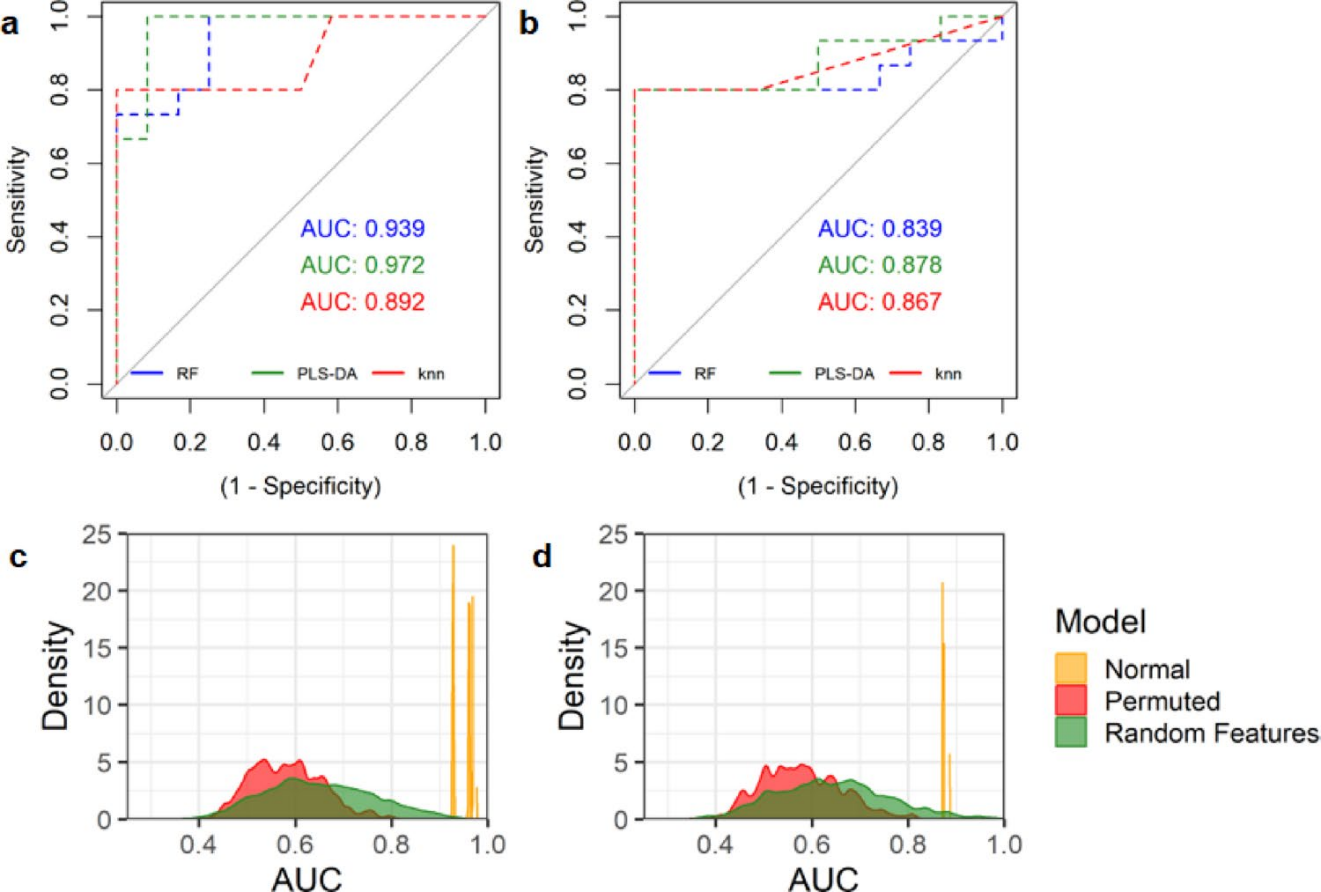
Prediction of CPE using annotated discriminatory metabolites reveals high predictive performance. Model performance depicted by ROC curves for RF, PLS-DA and kNN models based on endometabolome (**A**) and exometabolome (**B**) metabolites. PLS-DA endometabolome (**C**) and exometabolome (**D**) model performance was validated using permutation testing. Distribution of test set AUC values over 2000 iterations are displayed with regular model (yellow), model with permuted class labels (red), and randomly selected features (endometabolome *n* = 12, exometabolome *n* = 9) from the whole dataset with correct class labels (green)

To determine the ability of the metabolites to predict CPE, we combined the 12 endometabolome metabolites into single models using RF, PLS-DA and kNN algorithms which were tuned on training data. We tested the models on the unseen test set data over 2000 iterations and observed excellent performance metrics across all three machine learning algorithms on unseen test set data. The PLS-DA model displayed overall superiority for AUC (0.967) and specificity (81.7%), but was marginally lower than RF for sensitivity (RF = 100%, PLS-DA = 98.9%) (Fig. 4A; Table 3). Similarly. PLS-DA yielded the highest performance for the prediction of CPE using the nine putatively annotated exometabolome metabolites, although the metrics were lower than those of the endometabolome models (Fig. 4B; Table 3).

**Table 3:** Performance metrics for the prediction of CPE from MSI level 1 and 2 annotated metabolites using PLS-DA, kNN and RF machine learning models. Predictions were performed 2000 times against test set data. Metrics are displayed as endometabolome / exometabolome

	PLS-DA	kNN	RF
Specificity	0.817 / 1.000	0.504 / 0.713	0.749 / 0.915
Sensitivity	0.989 / 0.800	0.812 / 0.821	1.000 / 0.800
AUC	0.967 / 0.878	0.893 / 0.846	0.947 / 0.845

We next implemented permutation testing for comparison against the null hypothesis. Permutation of class labels over 2000 iterations of model testing was performed, resulting in a mean AUC of 0.582 for the endometabolome model, approximating random probability, compared to 0.967 (*p* < 0.05) achieved in 2000 iterations of the true model (Fig. 4C). For the exometabolome model, a mean AUC of 0.578 was observed after class label permutations compared to 0.878 (*p* < 0.05) in the real model (Fig. 4D). To assess overfitting, we randomly selected 12 features from the original unrefined endometabolome dataset and nine features from the exometabolome dataset during each of an additional 2000 iterations, resulting in mean AUCs of 0.650 and 0.643 (*p* < 0.05), respectively.

### Pathway analysis reveals mechanistic insights of resistant phenotype

To obtain further mechanistic insight into the pathways underlying the resistant phenotype, we investigated metabolite pathway enrichment within the complete endometabolome datasets. Pathway analysis of the entire complement of mass spectral data was performed using the mummichog algorithm with *Escherichia coli* K-12 MG1655 [KEGG] pathway library. CPE isolates displayed high topological significance and pathway enrichment values for amino acid, energy and sugar metabolism, and pathways related to the cell membrane including lipopolysaccharide biosynthesis, peptidoglycan biosynthesis, O-antigen nucleotide sugar biosynthesis, lipoic acid metabolism and fatty acid biosynthesis. Heme biosynthesis as well as redox regulatory pathways including glutathione metabolism and ubiquinone biosynthesis were also observed to be significantly enriched (Supplementary Fig. 4).

**Fig. 5:**
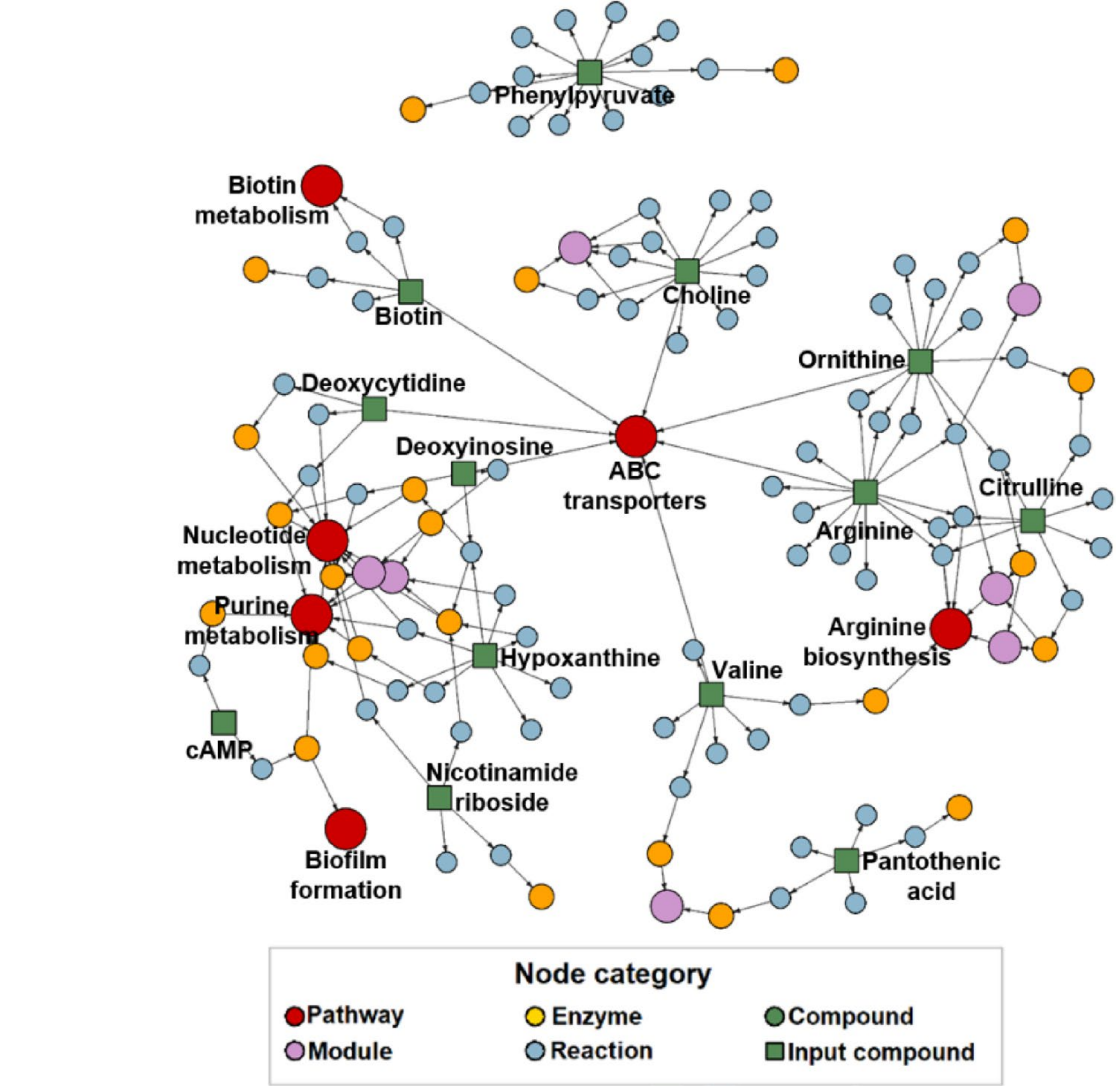
Pathway analysis reveals enriched microbial pathways. Node prioritisation results using FELLA pathway analysis with heat diffusion method. Green squares – annotated metabolites; red circles – pathways; pink circles – modules, yellow circles – enzymes, and blue circles – reactions

We next probed the pathways correlated with the annotated metabolites in the final models using FELLA (Picart-Armada et al., 2018) which performs network-based enrichment based on a set of implicated metabolites. Analysis revealed that the main pathways connecting the final model metabolites were arginine biosynthesis, purine metabolism, biotin metabolism, nucleotide metabolism, ATP-binding cassette (ABC) transporters and biofilm formation (Fig. 5).

## Discussion

Differences in metabolite levels between susceptible and resistant bacteria in the absence of antibiotics have been previously observed (Derewacz et al., 2013; Lin et al., 2019; Smart et al., 2019; Hewett et al., 2020; Ma et al., 2020; Mielko et al., 2021). Metabolic profiles have also been linked to susceptibility phenotype (Lin et al., 2019; Rego et al., 2021). Regarding carbapenem resistance in Enterobacterales, Rees et al. demonstrated that volatile metabolic fingerprints could be used to discriminate between CPE and non-CPE bacteria, and differential levels of metabolites have been observed in the plasma of patients with carbapenem-resistant *K. pneumoniae* infection (Rees et al., 2018; Wen et al., 2021). Furthermore, using NMR, Foschi et al. observed a range of differential metabolites between carbapenemase-producing and non-producing *K. pneumoniae* both with and without antibiotic stress (Foschi et al., 2018). On this basis, we hypothesised that the metabolome of CPE would differ from that of non-CPE, and that employing untargeted metabolomics using high resolution LC-MS could elucidate discriminative metabolites. By analysing the metabolomic signatures of CPE using liquid chromatography-mass spectrometry and multivariate analysis, we aimed to identify discriminative metabolites which could be exploited for the modelling and prediction of CPE. We believe this to be the first study to utilise LC-MS for the investigation of the CPE metabolome.

We observed significant differences in the metabolomes of CPE compared to non-CPE bacteria. Using three machine learning algorithms, we performed robust feature selection and the resulting panel of 21 metabolite biomarkers showed high predictive performance across all models (AUC = 0.845–0.967). In addition to parental *m/z* detection, we performed MS/MS on an inclusion list of discriminatory mass features to obtain putative compound annotations. Metabolites in our final models met the following selection criteria: (a) Bonferroni-corrected *p*-value ≤ 0.05, (b) high variable importance in at least two supervised models, and (c) putatively annotated with a biologically relevant compound. Consequently, 12 endometabolome and nine exometabolome metabolites were selected as robust indicators of resistance. We were able to confirm 14 of these to MSI level 1 matching with authentic standards.

Many of our models’ constituent metabolites are implicated in pathways associated with cofactor and vitamin metabolism. Pyridoxamine (one of six vitamin B_6_ vitamers), for example, has been shown to be involved in the salvage of pyridoxal 5’-phosphate, the biochemically active form of vitamin B_6_, which is a coenzyme in a variety of reactions (Tramonti et al., 2021). Furthermore, it has been linked to pathways associated with microbial metabolism in diverse environments (Kanehisa & Goto, 2000). Similarly, pantothenic acid (vitamin B_5_) is established as an essential precursor for the biosynthesis of coenzyme A and plays a role in membrane lipid metabolism, whilst biotin (vitamin B_7_) is the essential cofactor of biotin-dependent carboxylases, such as acetyl-CoA carboxylase where it is required in the first step of fatty acid biosynthesis (Cronan & Waldrop, 2002; Leonardi & Jackowski, 2007). Biotin is not synthesised *de novo* in mammals, making its associated pathways attractive targets for antibiotic discovery and targeting (Sirithanakorn & Cronan, 2021). Nicotinamide riboside, meanwhile, is a form of vitamin B_3_, and functions as a precursor to nicotinamide adenine dinucleotide (NAD+). NAD + serves as a co-enzyme for a broad range of physiological processes, including the maintenance of redox homeostasis and genomic stability (Chen et al., 2024). Aside from pyridoxamine, all cofactor and vitamin-associated metabolites which were annotated from our datasets were observed to be higher in the CPE group.

Higher levels of the complex formed between cAMP and the cAMP receptor protein (CRP) have been previously observed with increasing antibiotic resistance to ampicillin, another β-lactam antibiotic (Jiang et al., 2023). Our results are consistent with these findings and revealed higher levels of cAMP in CPE isolates. It is thought that the cAMP-CRP complex plays a role in the acquisition of resistance through inactivation of DNA repair, increasing the mutation rate, not dissimilar to that observed with the overexpression of AcrAB (El Meouche & Dunlop, 2018). Interestingly, cAMP-CRP also mediates biofilm formation in both *E. coli* and *K. pneumoniae* (Liu et al., 2020). Through activation of curli fibres expression and flagellum biosynthesis, coupled with repression of the stress responses which inhibit the initiation of biofilm formation, cAMP-CRP plays a role in the positive regulation of biofilm formation (Ou et al., 2017; Jiang et al., 2023).

Lastly, carbapenem antibiotics target bacterial cell wall biosynthesis, resulting in its inhibition. In prokaryotes, UDP-glucose is a common precursor for lipopolysaccharide O-antigens and cell wall polymers, including peptidoglycan (Garde et al., 2021). We observed higher levels of UDP and UDP-glucose in the CPE group, as well as enrichment of pathways related to the cell membrane and wall, including lipopolysaccharide biosynthesis, peptidoglycan biosynthesis, O-antigen nucleotide sugar biosynthesis, lipoic acid metabolism and fatty acid biosynthesis.

Pathway analysis of these metabolites revealed high enrichment values for arginine biosynthesis, purine metabolism, biotin metabolism, ABC transporters, biofilm formation and nucleotide metabolism. Recent studies into the metabolic reprogramming of *E. coli* and *K. pneumoniae* isolates after multidrug resistant plasmid acquisition suggest that the acquisition of such a plasmid not only confers antibiotic resistance but also alters global gene expression (Long et al., 2019; Carrilero et al., 2023; Hall et al., 2024). It has been proposed that plasmid acquisition may elicit evolutionary responses within the bacterial genome that facilitate the integration and stable maintenance of the plasmid itself. Interestingly, a link has already been demonstrated between arginine metabolic pathways and the acquisition of plasmids harbouring multidrug resistance genes, including carbapenemases (Carrilero et al., 2023; Hall et al., 2024). We observed that CPE isolates had higher levels of ornithine and lower levels of arginine in the extracellular matrix. The uptake of arginine and secretion of ornithine by pathogenic enteric bacteria may enhance toxin production, upregulate virulence genes, stimulate growth and biofilm production, as well as promote other pathobionts (Keogh et al., 2016; Nüse et al., 2023; Lillie et al., 2024). The depletion of extracellular arginine as an immune evasion mechanism has been well established, leading to reduced host nitric oxide synthesis and dysregulated immune response (Das et al., 2010). Thus, whilst arginine metabolism might not be directly linked to carbapenemase production, it may influence colonisation and bacterial persistence, modulating the host-pathogen response. In addition, studies have also reported gene enrichment of ABC transporters in multidrug resistant bacteria, although this was found to be plasmid-specific (Long et al., 2019; Carrilero et al., 2023; Hall et al., 2024). In one instance, the commonly mutated gene after clonal evolution both with and without antibiotic stress encoded part of the arginine ABC transporter complex, demonstrating parallel evolution across strains (Carrilero et al., 2023). Further investigation into the association between arginine and ABC transporter pathways and the acquisition of multidrug resistance is warranted to understand the underlying cause. Furthermore, deciphering the metabolic reprogramming in which pathogens harbouring these resistant genes undergo may elucidate potential drug targets in the fight against antimicrobial resistance.

In summary, we have demonstrated the ability to model CPE based on the metabolome using high resolution LC-MS/MS. We have unveiled potential metabolite biomarkers with diagnostic potential for the presence of CPE with high predictive performance, and highlighted pathways likely to be contributing to the overall resistance phenotype. Future studies should seek to validate these metabolites and ascertain whether the observed metabolic signatures continue to be seen in clinically relevant matrices such as blood and urine, as we acknowledge the in vitro nature of our experimental set up and the metabolic variation that exists between matrices. Furthermore, pathway perturbation experiments may reveal whether these differential metabolites are resultant of carbapenemase production or arise from other means such as plasmid compensatory mechanisms. Together, our results show that metabolomics analysis of CPE can provide deeper mechanistic insight into the carbapenem-resistant phenotype, offering information which may not be revealed by other–omics investigations.

## Methods and materials

### Bacterial isolates

*E. coli* (*n* = 14) and *K. pneumoniae* (*n* = 18) isolates were used in the study. The strains were genotypically categorised as CPE (*n* = 18) or non-CPE (*n* = 14) based on genome sequencing data and phenotypically characterised as susceptible (S), susceptible – increased exposure (I) or resistant (R) to meropenem by antimicrobial susceptibility testing based on broth microdilution and Kirby-Bauer tests. All strains were clinical isolates donated by the North Bristol NHS Trust and Manchester University NHS Foundation Trust with the exception of those obtained from the National Collection of Type Cultures (UK Health Security Agency, UK): *K. pneumoniae* NCTC 13438, ATCC BAA-1705, ATCC 13883, ATCC 13887, NCTC 13442 and NCTC 13443; *E. coli* NCTC 14325, NCTC 14339 and ATCC 25922. A non-pathogenic laboratory *E. coli* BL21(DE3) strain was obtained from New England BioLabs (Massachusetts, USA).

### Antibiotic susceptibility testing

Susceptibility testing was performed according to the 2022 European Committee on Antimicrobial Susceptibility Testing (EUCAST) guidelines using the Kirby-Bauer method (EUCAST, 2022c). The following antibiotic disks were used: 10 µg meropenem, 10 µg imipenem, 5 µg cefotaxime and 20/10 µg amoxicillin-clavulanic acid (Oxoid, UK). In addition, 30 µg cefotaxime (Oxoid, UK) and 20/10 µg amoxicillin-clavulanic acid utilised as a double disk synergy test with 20 mm centre-to-centre separation to assess ESBL-production. Zone of inhibition diameters were recorded after 18 h incubation at 35 °C. Three independent experiments of each assessment were performed. Results were validated using the American Type Culture Collection (ATCC) quality control strain *E. coli* ATCC 25922. Measurements were compared to EUCAST breakpoints to characterise strains as susceptible (S), susceptible – increased exposure (I) or resistant (R) (EUCAST, 2021).

The broth microdilution method was implemented for determination of the meropenem minimum inhibitory concentration (MIC) (EUCAST, 2022b). Each strain was assayed in triplicate across a range of 0.004–128 mg L^− 1^ meropenem (meropenem trihydrate; APExBIO Technology, USA). The method was performed three times across different days. Results were interpreted using the 2022 EUCAST breakpoint criteria (EUCAST, 2022a).

### Whole genome sequencing and genomic analysis

Whole genome sequencing was performed by MicrobesNG, United Kingdom. Cells were harvested from pure liquid cultures via centrifugation and resuspended in DNA/RNA Shield (Zymo Research, USA) before being sent to the sequencing facility. DNA libraries were prepared using the Nextera XT Library Prep Kit (Illumina, USA) following the manufacturer’s protocol with the following modifications: input DNA was increased 2-fold, and PCR elongation time was increased to 45 s. Pooled libraries were quantified using the Kapa Biosystems Library Quantification Kit for Illumina. Libraries were sequenced using Illumina sequencers (HiSeq/NovaSeq) using a 2 × 250 bp paired-end protocol. Reads were adapter trimmed using Trimmomatic v0.30 with a sliding window quality cut-off of Q15 (Bolger et al., 2014). De novo assembly was performed on samples using SPAdes version 3.7 (Bankevich et al., 2012), and contigs were annotated using Prokka 1.11 (Seemann, 2014).

Species identification was performed using SpeciesFinder 2.0 (Larsen et al., 2014). Multilocus sequence typing (MLST) analysis was performed on seven housekeeping genes (*K. pneumoniae: gapA*,* infB*,* mdh*,* pgi*,* phoE*,* rpoB*,* tonB; E. coli: adk*,* fumC*,* gyrB*,* icd*,* mdh*,* purA*,* recA*) using MLST 2.0 (Larsen et al., 2012).

Antimicrobial-resistance genes (ARGs) and chromosomal point mutations conferring AMR were identified using ResFinder 4.1 (Bortolaia et al., 2020); hits with ≥ 95% identity and ≥ 90% coverage were accepted. Identifications were cross-checked using the Comprehensive Antibiotic Resistance Database (CARD) (Alcock et al., 2023).

### Preparation of cultures for untargeted metabolomics

Glycerinated stocks of each isolate were streaked onto tryptic soy agar plates and grown overnight for 18 h at 37 °C. Single homologous colonies were inoculated in tryptic soy broth (TSB) (Sigma Aldrich) and grown for a further 18 h to produce overnight pre-cultures. Three independent biological replicates were inoculated from each pre-culture in 10 mL TSB to give a starting OD_600_ of 0.01. Cultures were grown to late log phase (6 h; 37 °C with 180 rpm shaking). TSB-only aliquots were prepared concurrently and subjected to the same conditions. Following incubation, OD_600_ values were measured for normalisation during later reconstitution and can be found in Supplementary Table 1.

### Exometabolome metabolite sample Preparation

A 2 mL aliquot from each culture was removed for exometabolome analysis. Aliquots were centrifuged (10 min; 10,000 *g*), and supernatant was filtered through a 0.2 μm syringe filter (Sartorius). Media-only preparations were treated to an identical process. An 80 µL aliquot of each filtrate was evaporated under vacuum using an Eppendorf Concentrator Plus and the dried pellet was stored at − 80 °C until analysis.

### Endometabolome metabolite extraction

Bacterial metabolism was quenched by rapidly combining incubated cultures (5 mL) with 2:1 *v/v* -48 °C 60% methanol. Resulting samples were centrifuged (10 min; 3,200 *g* and − 9 °C), after which the supernatant was discarded.

Quenched pellets were resuspended in 1.5 mL of 2:1 *v/v* CHCl_3_:MeOH (-20 °C). Suspensions were transferred to fresh Eppendorf tubes and vortexed for 5 min. A 500 µL aliquot of cold (4 °C) LC-MS-grade water was added, and the contents were vortexed for a further 30 s. The samples were then centrifuged (3 min; 13,300 *g* and 4 °C). The upper polar layer was transferred to a fresh tube and centrifuged (5 min; 16,500 *g* and 0 °C). The remaining non-polar bottom layer was then centrifuged (3 min; 13,300 *g* and 4 °C). An 800 µL aliquot of each extract was transferred to a fresh tube and evaporated under vacuum centrifugation. The dried pellet was stored at − 80 °C until analysis.

### Data acquisition

Frozen pellets were reconstituted in 60% *v/v* methanol (100 µL and 500 µL per OD_600_ of 0.74 for endometabolome extracts and exometabolome samples, respectively), vortex mixed and centrifuged (3 min; 13,400 *g*). Pooled QC samples were prepared by combining 5 µL of each sample within each sample group e.g. exometabolome, non-polar endometabolome and polar endometabolome. Samples were transferred to low-recovery mass spectrometry vials and were analysed within 72 h of reconstitution with storage in the autosampler at 4 °C during analysis.

LC-MS/MS analysis was performed using a Q Exactive™ Plus Orbitrap™ mass spectrometer (ThermoFisher Scientific, UK) with a heated electrospray ionisation (HESI) ion source. Ion source parameters were: spray voltages, + 3.5 and − 4 kV for positive- and negative-mode, respectively; sheath N_2_ gas flow, 50 AU; aux N_2_ gas flow, 13 AU; sweep N_2_ gas flow, 3 AU; capillary temperature, 320 °C; aux gas temperature, 425 °C; S-lens RF level, 50%. Full scan MS spectra were acquired over the range of *m/z* 90–1350 at a mass resolution of 70,000, with AGC target of 1e^6^ and a maximum injection time of 100 ms. Selected features of interest were compiled into an inclusion list for selective ion monitoring (SIM) of QC samples, with data dependant acquisition ddMS^2^ performed on matched precursor ions at a resolution of 35,000. A stepped higher-energy collisional dissociation (HCD) approach was implemented at levels of 15, 35 and 70 AU, as well as 25, 50 and 100 AU.

For both reverse-phased (RP) and hydrophilic interaction liquid chromatography (HILIC) chromatography, 2 µL of each sample was injected into an UltiMate3000 UHPLC. Mobile phase was delivered at 300 µL min^− 1^ and UHPLC columns were maintained at 45 °C. For reverse-phased analysis, an Accucore™ Vanquish™ C18 + UHPLC column (2.1 × 100 mm, 1.5 μm; ThermoFisher Scientific, UK) was used. Eluent A and B comprised of LC-MS grade water and methanol, respectively, both containing 0.1% *v/v* formic acid. Gradient elution was performed in both ion modes with the following profile: 0–1 min: 2% B, 1–11 min: 2–100% B, 11–14 min: 100% B, 14–14.01 min: 100–2% B, 14.01–17 min: 2% B. For HILIC analysis, a SeQuant^®^ ZIC^®^-cHILIC column (2.1 × 100 mm, 3 μm, 100 Å; Merck, UK) was selected. Eluent A and B comprised of LC-MS grade water and acetonitrile, respectively, both containing 0.1% *v/v* formic acid. The gradient elution profile was as follows: HESI + 0–13 min: 98–20% B, 13–13.01 min: 20–98% B, 13.01–17 min: 98% B; HESI- 0–5 min: 98–70% B, 5–10 min: 70–20% B, 10–10.01 min: 20–98% B, 10.01–15 min: 98% B. Non-polar and polar extracts were run on RP and HILIC methods, respectively, while exometabolome samples were analysed on both methods. For each sample, data were acquired in both positive (ESI+) and negative (ESI-) ion-mode.

### Standard confirmation

Pyridoxamine (pyridoxamine dihydrocholride; Tokyo Chemical Industry), biotin (Sigma-Aldrich), UDP (uridine 5’-diphosphate sodium salt hydrate; Cayman Chemical Company), UDP-glucose (uridine 5’-diphosphoglucose disodium salt hydrate; Sigma-Aldrich), phenylpyruvate (phenylpyruvic acid; Fluorochem), L-citrulline (Sigma-Aldrich), cAMP (Tokyo Chemical Industry), pantothenic acid (D-calcium pantothenate; ThermoFisher Scientific), choline (choline chloride; ThermoFisher Scientific), ornithine (L-ornithine hydrochloride; ThermoFisher Scientific), deoxycytidine (2’-deoxycytidine hydrochloride; ThermoFisher Scientific) and arginine (L-arginine; MP Biomedicals) were prepared at 1 mg mL^−1^ in LC-MS grade water. Deoxyinosine (2’-deoxyinosine; ThermoFisher Scientific) was prepared at 1 mg mL^−1^ in LC-MS grade methanol, whilst hypoxanthine (Fluorochem) was prepared in 2:1 formic acid:water. Standards were analysed with LC-MS using a single ion monitoring (SIM) method based on the precursor *m/z* values of the MSI level 2 annotated metabolites, with identical LC-MS parameters to those outlined previously. MS/MS spectra were compared against those generated for the putatively annotated metabolites for confirmation of annotations to MSI level 1.

### Data preprocessing

Raw data files were centroided and converted to mzML format using MSConvert (Kessner et al., 2008). Spectral preprocessing was undertaken using the package XCMS (v3.22.0) in R v4.3.1 with standard procedures (Smith et al., 2006; R Core Team, 2022). XCMS parameter optimisation was performed with the package IPO (v1.26.0) on the 24 QC samples of each analytical run (Libiseller et al., 2015). The selected XCMS parameters for all ion-mode and stationary phase combinations of the endo- and exometabolome analyses are found in Supplementary Tables 2 and 3, respectively. Peak matrix processing was undertaken on each dataset using the R package pmp (v1.12.0) (Jankevics et al., 2023). Data were filtered such that features were removed if (a) peaks were missing in more than 25% of pooled QC samples, (b) in the remaining peaks, if RSD of peak intensities across pooled QC samples was > 20%, and (c) finally, from remaining peaks if missing values (NaN) were observed in > 80% of all the samples. Background subtraction was performed on exometabolome datasets by subtracting peaks in the batch media samples from the experimental samples belonging to the same batch.

In the final feature list, missing values were replaced using the small value imputation method, whereby half of the lowest value recorded in the entire data matrix was added in place of a zero. Probabilistic quotient normalisation (PQN) was performed, followed by glog transformation and pareto scaling prior to multivariate analyses. Putative annotation was performed using tandem mass spectrometry data. MS/MS spectra were matched against GNPS’ spectral libraries using GNPS MASST and Molecular Library Search with default parameters (minimum cosine value of 0.7, parent mass tolerance of 2 Da and fragment tolerance of 0.5 Da) (Wang et al., 2016). Similarity searches were performed against spectra within the MassBank of North America (MoNA) database with minimum similarity 600. Tandem mass spectral peak lists were also searched against the *E. coli* Metabolome Database (ECMDB) 2.0 with precursor tolerance of 0.05 Da (Sajed et al., 2016).

## Statistical analysis and machine learning modelling

### Univariate comparisons

The mean intensities for each mass feature measured in CPE and non-CPE samples were statistically evaluated using Welch’s t-test (*p-*value ≤ 0.05). The Bonferroni correction was then applied to *p*-values, with *q* ≤ 0.05 considered significant.

### Multivariate analyses

Three multivariate classification algorithms (partial least squares-discriminant analysis (PLS-DA), *k*-nearest neighbours (kNN), and random forest (RF) were implemented using the caret package (v6.0.94) in R for the prediction of CPE versus non-CPE (Kuhn, 2008). Datasets were split 70:30 into training and test sets, with stratified but random assignment. Classifier models were trained on the complete set of training data with 10 times repeated 5-fold cross-validation. Variable importance to each model was calculated for the purposes of feature selection using the average feature rank across all discovery-validation iterations. Models were retuned on the training set data using only the top discriminatory features. The retuned models were used to predict the class of test set samples. The top discriminatory metabolites for an algorithm were identified. Receiver operating characteristic (ROC) curves were generated using the average test set class probabilities for each sample. Area under the ROC curve (AUC), as well as accuracy, specificity and sensitivity were used as performance metrics to evaluate the diagnostic power of the models over 2000 iterations.

### Permutation testing

For comparison against the null distribution, models were tested over 2000 iterations, during which, class labels of test set samples i.e. “CPE” and “non-CPE” were randomly permuted. To assess overfitting, 12 features from the original unrefined endometabolome dataset and nine features from the exometabolome dataset were randomly selected during model training over each of 2000 iterations. AUC values of test set data were used to assess model performance.

### Pathway analysis

Pathway analysis of the entire complement of mass spectral data was performed using the functional analysis module of MetaboAnalyst 5.0 based on the mummichog algorithm with *Escherichia coli* K-12 MG1655 [KEGG] pathway library with *m/z* values, retention times and *p*-values (Kanehisa & Goto, 2000; Pang et al., 2021; Lu et al., 2023). Pathway enrichment analysis of the annotated metabolites was performed using the R package FELLA (v1.20.0) (Picart-Armada et al., 2018). A KEGG graph and local database were derived from the KEGG ‘eco’ database (Release 109.0, accessed February 16th 2024), to which the annotated metabolites from the final models were mapped. Data were then enriched using the PageRank method for node scoring with *z*-score normalisation to reduce bias. Node prioritisation was performed using a *p*-value cut-off of 0.05 and node limit of 150.

## Electronic supplementary material

Below is the link to the electronic supplementary material.

Supplementary Material 1

## Data Availability

Raw spectral data files and study information are available in the MetaboLights database (Yurekten et al., 2024) under study ID MTBLS10129 (https://www.ebi.ac.uk/metabolights/MTBLS10129/). Whole genome sequencing data were deposited in the NCBI genome database under BioProject ID PRJNA1134938. The sequencing data for EC026 is available from BioProject PRJNA20713, BioSample SAMN02603478.
