# Supplementary material for: LC-MS/MS metabolomics unravels the resistant phenotype of carbapenemase-producing Enterobacterales
Source: Metabolomics. 2025 Aug 12;21(5):115. doi: 10.1007/s11306-025-02300-9 (PMC12343662; doi:10.1007/s11306-025-02300-9)
Supplement: Supplementary file 1 — Supplementary Material 1 [file 11306_2025_2300_MOESM1_ESM.pdf]

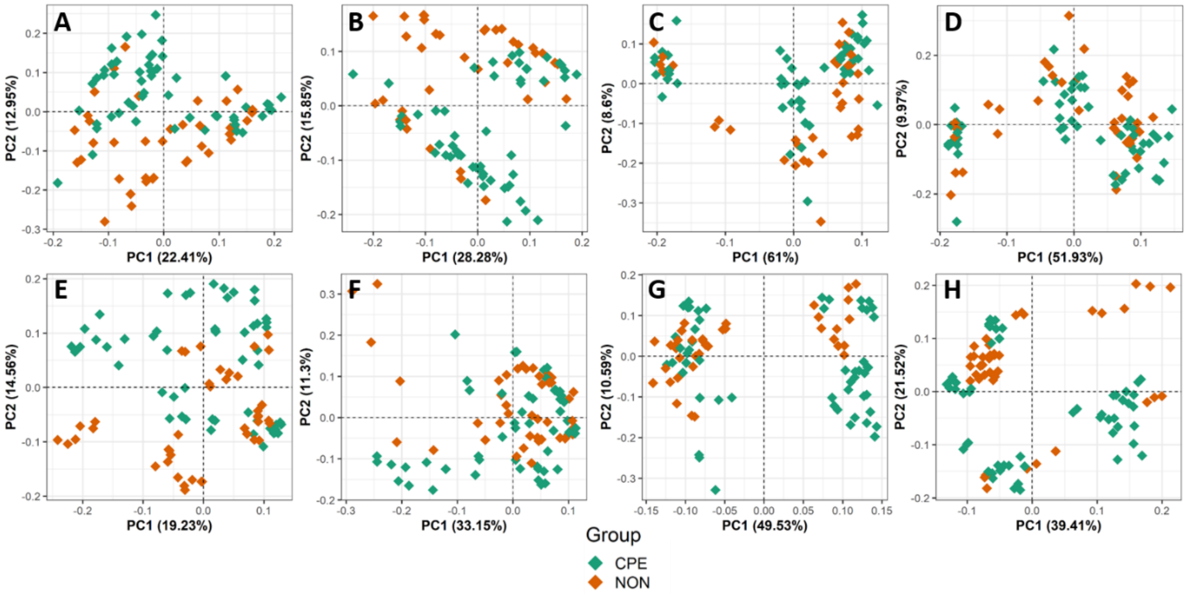

Supplementary Figure 1. CPE unable to be distinguished from non-CPE by unsupervised multivariate analysis. Principal component analysis (PCA) scores plots generated using all detected endometabolome **(A-D)** and exometabolome **(E-H)** features for the comparison of CPE (green) and non-CPE (red). Data obtained using LC-MS with HILIC ESI+ **(A and E)**, HILIC ESI- **(B and F)**, RP ESI+ **(C and G)** and RP ESI- **(D and H)** modes.

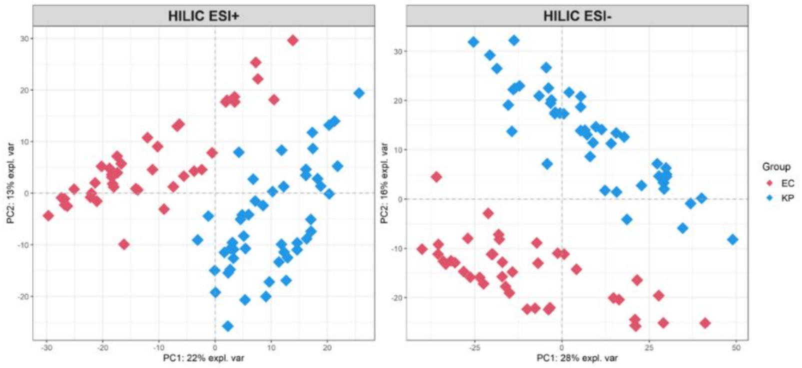

Supplementary Figure 2. Principal component analysis of endometabolome HILIC-LC-MS data grouped by species.

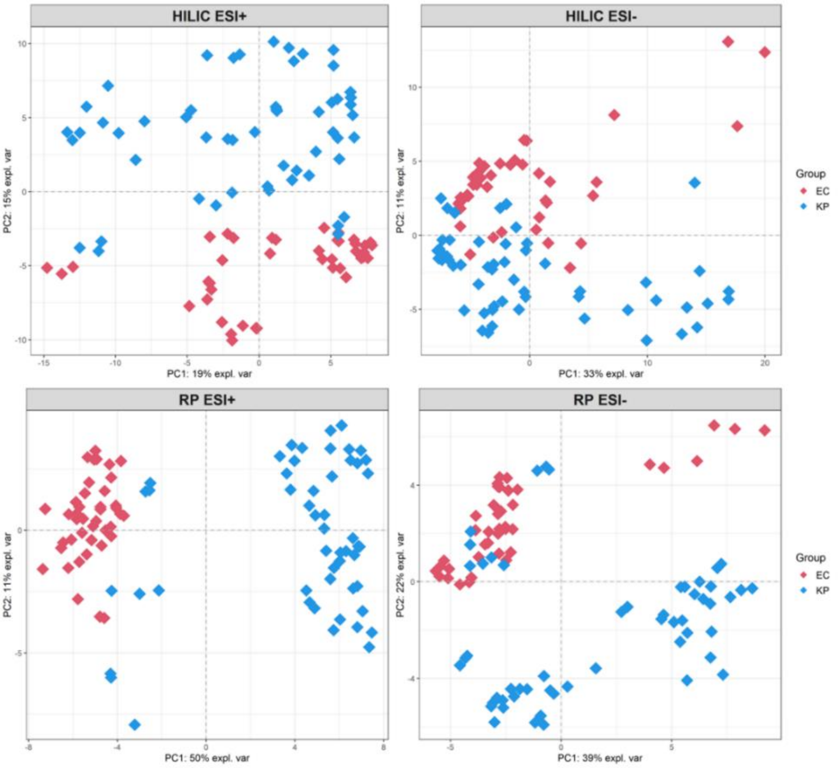

Supplementary Figure 3. Principal component analysis of exometabolome LC-MS data grouped by species.

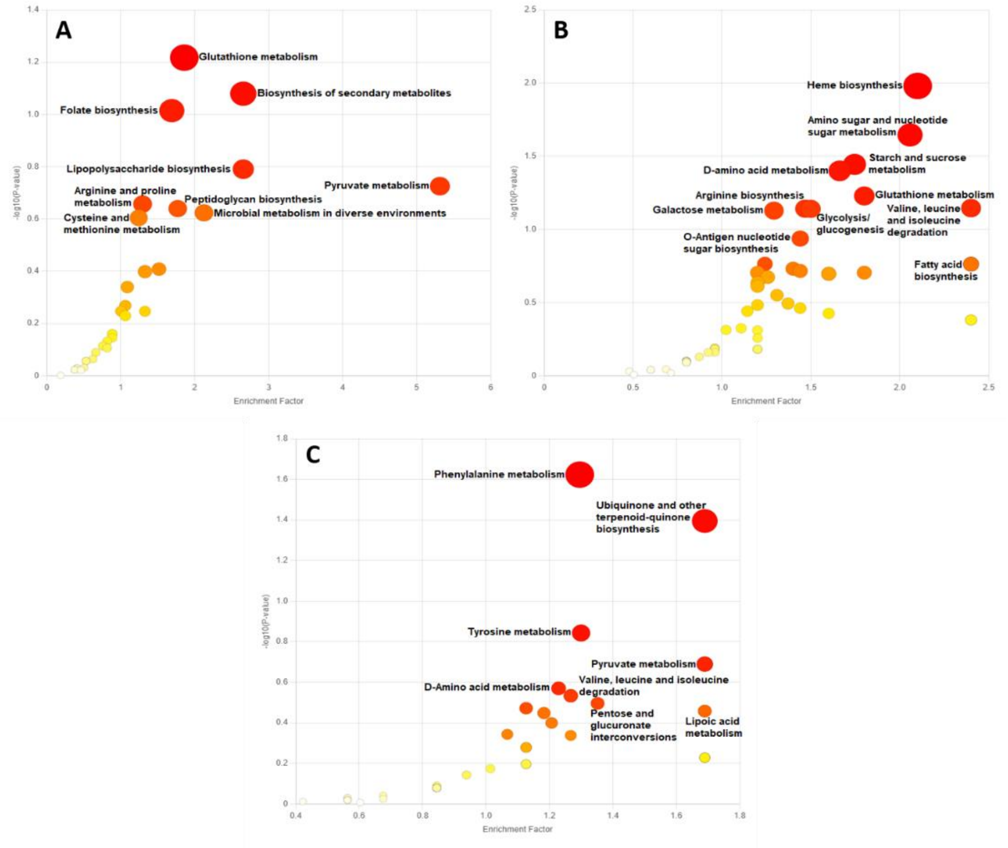

Supplementary Figure 4. Functional analysis results of features from intracellular A) HILIC ESI+, B) HILIC ESI- and C) RP ESI+ complete datasets. Pathway enrichment analysis performed using *m/z* and retention time based on mummichog algorithm with *Escherichia coli* K-12 MG1655 [KEGG] pathway library. RP ESI- contained no significant hits.

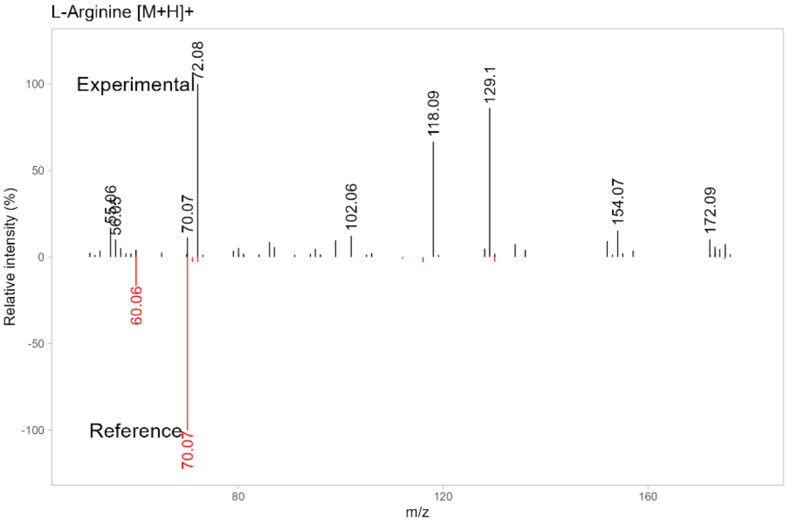

Supplementary Figure 5. MS/MS spectral match for L-arginine against standard reference compound.

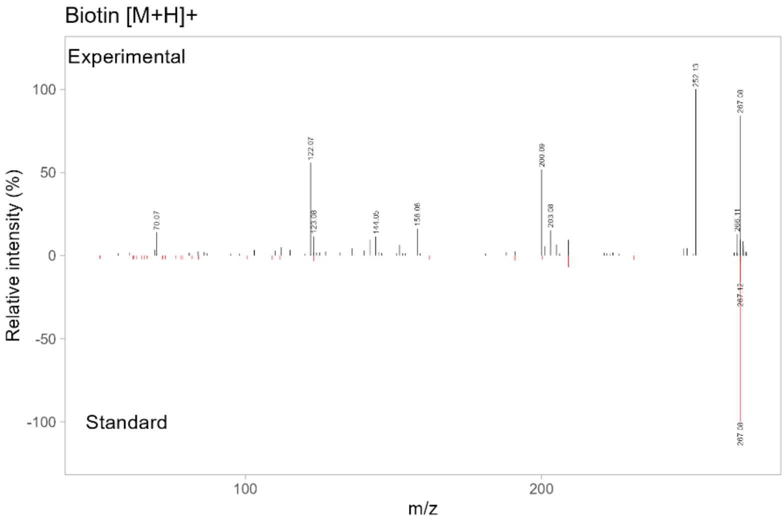

Supplementary Figure 6. MS/MS spectral match for biotin against standard reference compound.

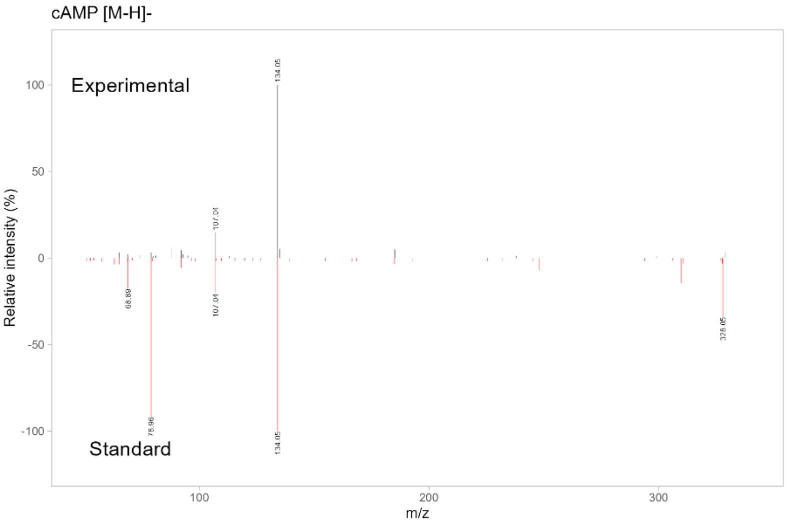

Supplementary Figure 7. MS/MS spectral match for cAMP against standard reference compound.

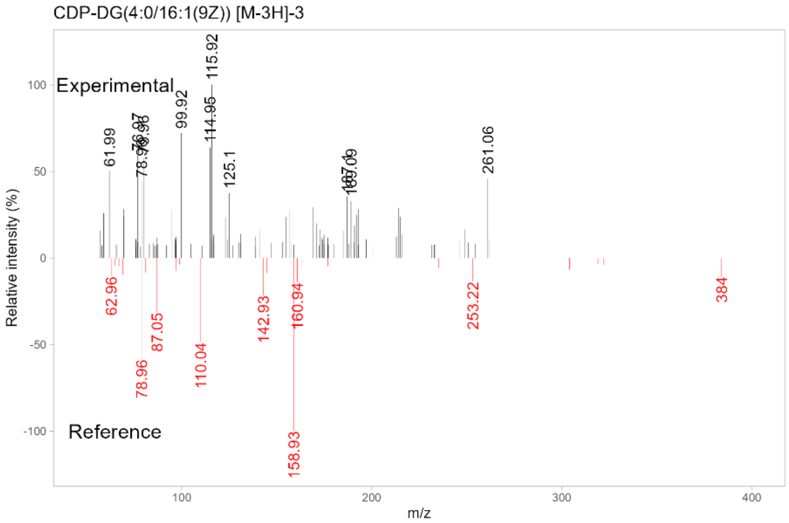

Supplementary Figure 8. MS/MS spectral match for CDP-DG(4:0/16:1(9Z)) against spectral library using MassBank of North America. Reference spectrum ID: splash10-029i-9411110000-850a97bda88f98d2dd1e.

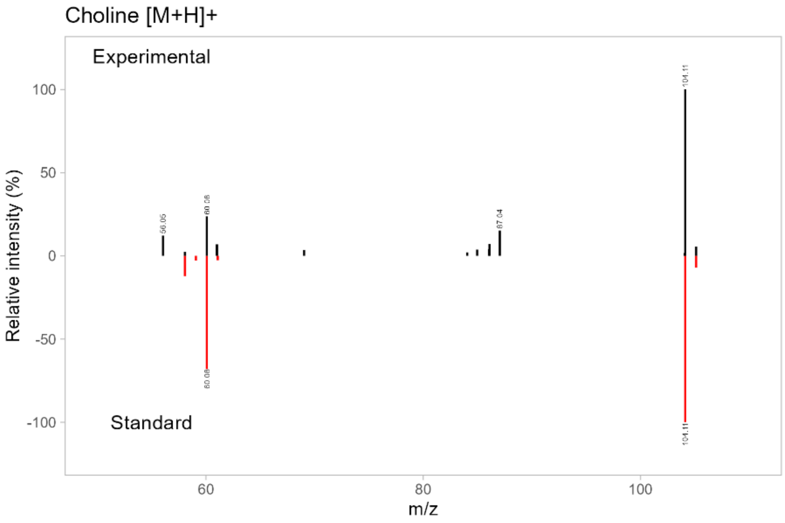

Supplementary Figure 9. MS/MS spectral match for choline against standard reference compound.

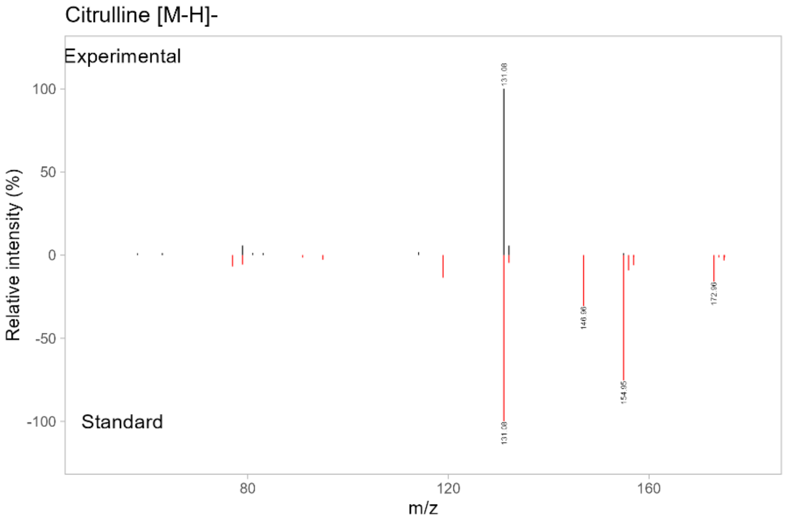

Supplementary Figure 10. MS/MS spectral match for L-citrulline against standard reference compound.

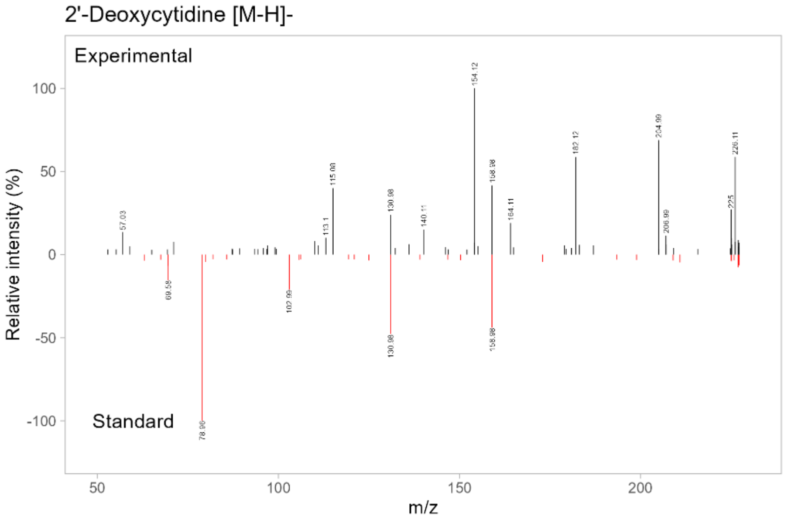

Supplementary Figure 11. MS/MS spectral match for 2’-deoxycytidine against standard reference compound.

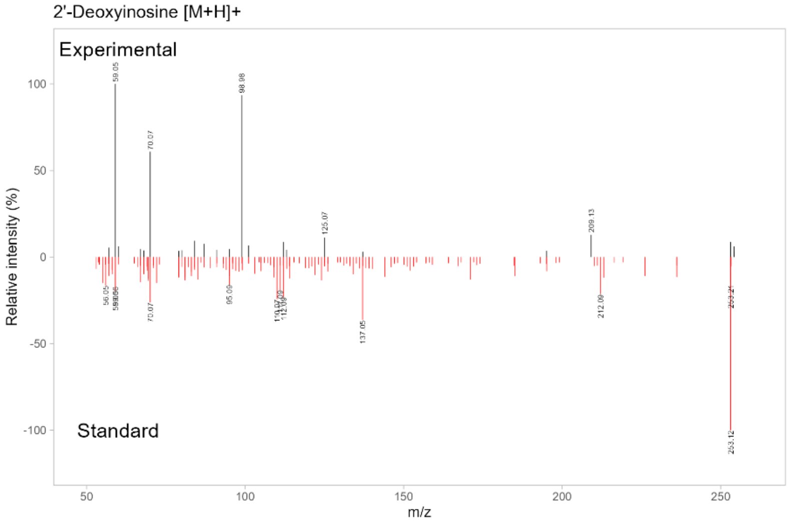

Supplementary Figure 12. MS/MS spectral match for 2’-deoxyinosine against standard reference compound.

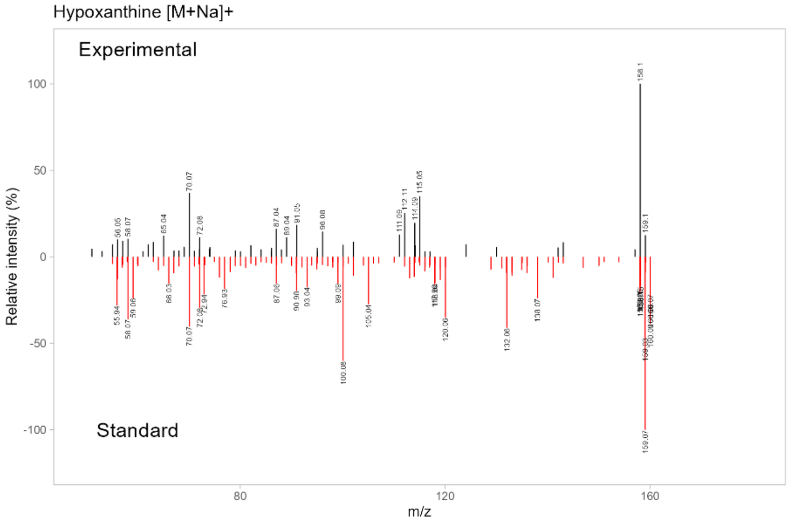

Supplementary Figure 13. MS/MS spectral match for hypoxanthine against standard reference compound.

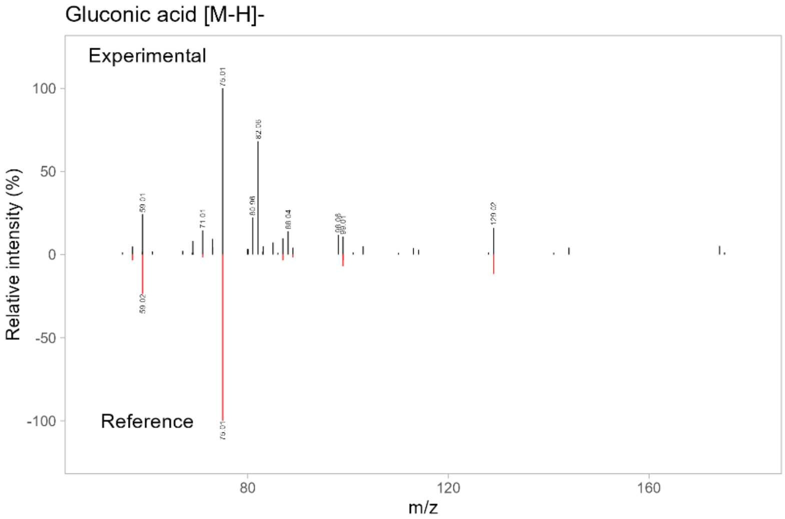

Supplementary Figure 14. MS/MS spectral match for gluconic acid against spectral library using MassBank of North America. Reference spectrum ID: splash10-004i-9200000000-626c31e8e20ed46540e2.

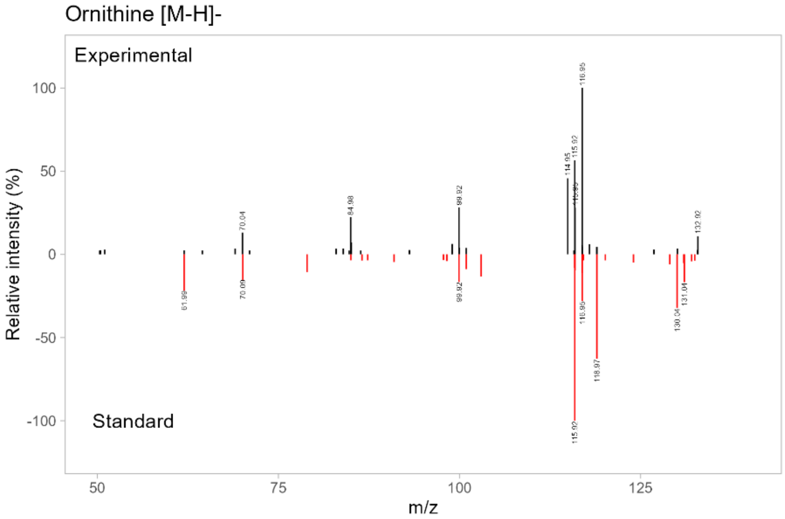

Supplementary Figure 15. MS/MS spectral match for ornithine against standard reference compound.

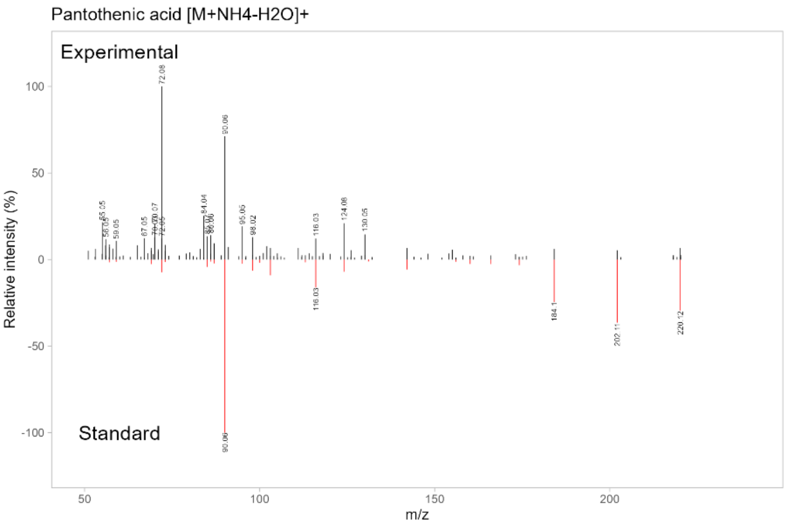

Supplementary Figure 16. MS/MS spectral match for pantothenic acid against standard reference compound.

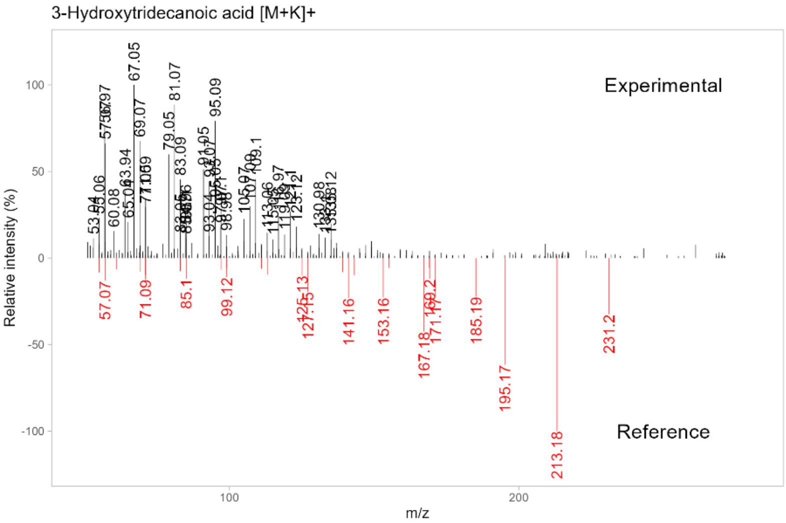

Supplementary Figure 17. MS/MS spectral match for 3-hydroxytridecanoic acid against spectral library using MassBank of North America. Reference spectrum ID: splash10-03dj-4940000000-e961beef0102305256ae.

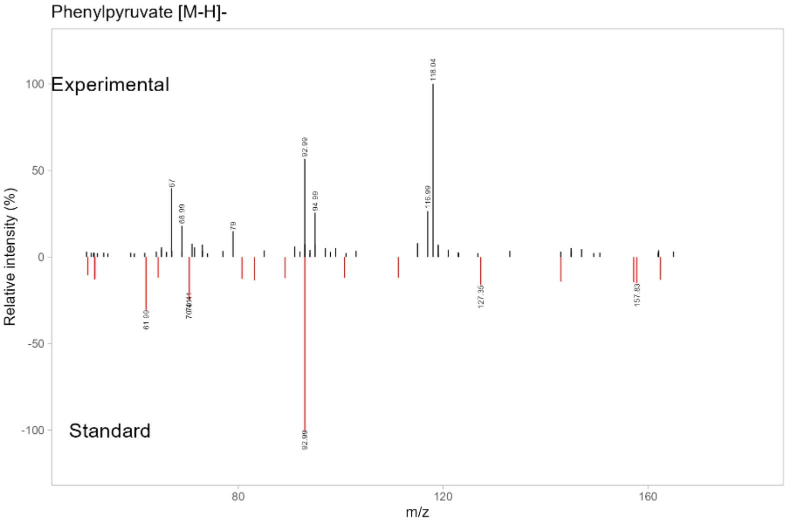

Supplementary Figure 18. MS/MS spectral match for phenylpyruvate against standard reference compound.

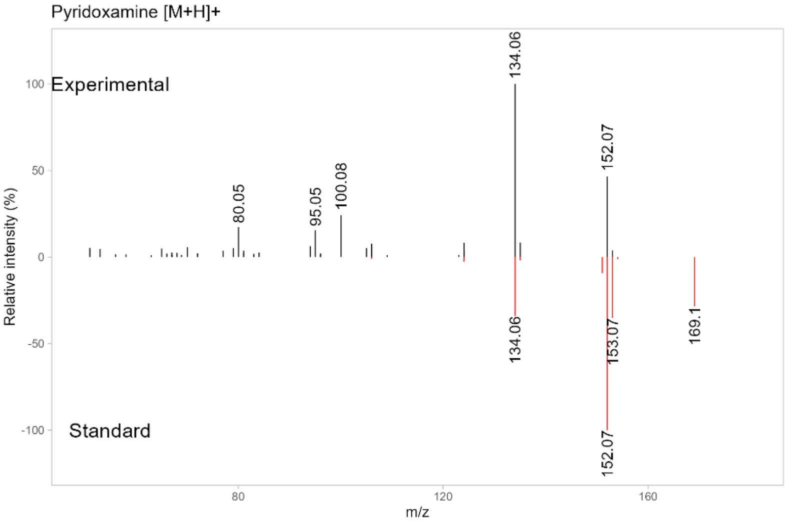

Supplementary Figure 19. MS/MS spectral match for pyridoxamine against standard reference compound.

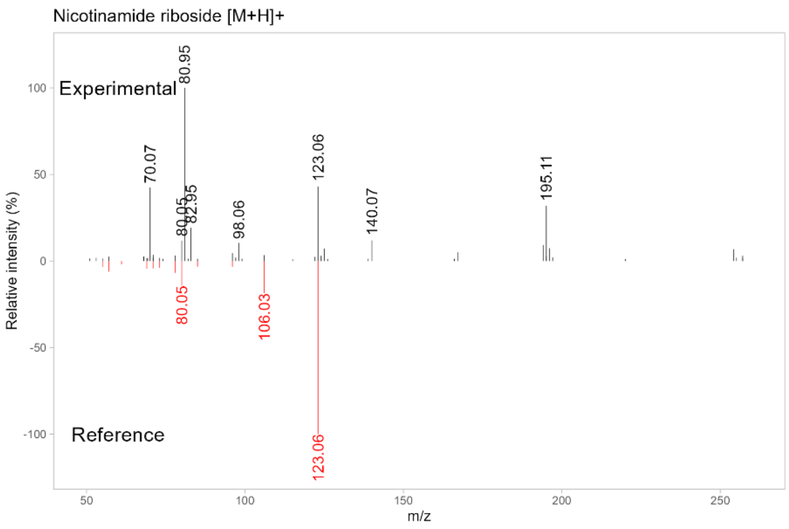

Supplementary Figure 20. MS/MS spectral match for nicotinamide riboside against spectral library using MassBank of North America. Reference spectrum ID: splash10-00di-3900000000-cd1f66bfc8ded7bd098e.

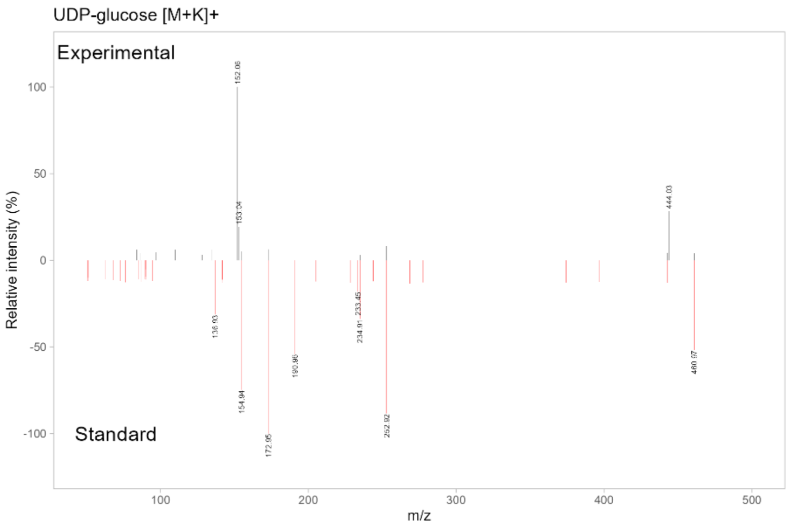

Supplementary Figure 21. MS/MS spectral match for UDP-glucose against standard reference compound.

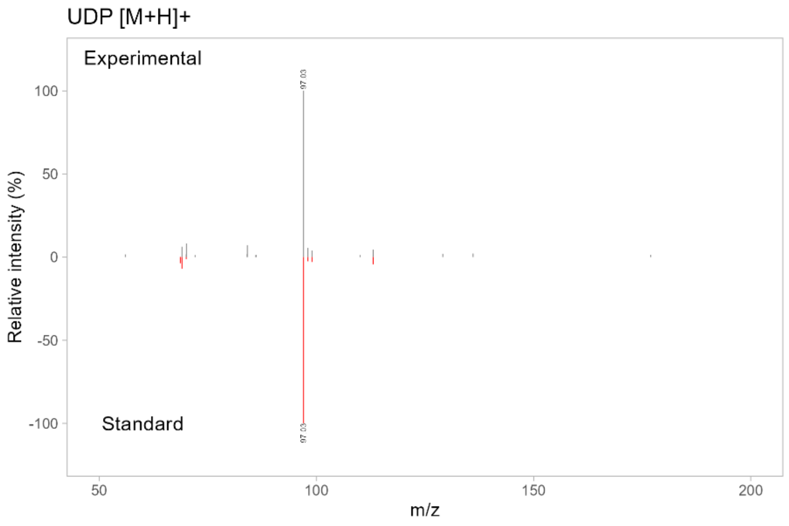

Supplementary Figure 22. MS/MS spectral match for UDP against standard reference compound.

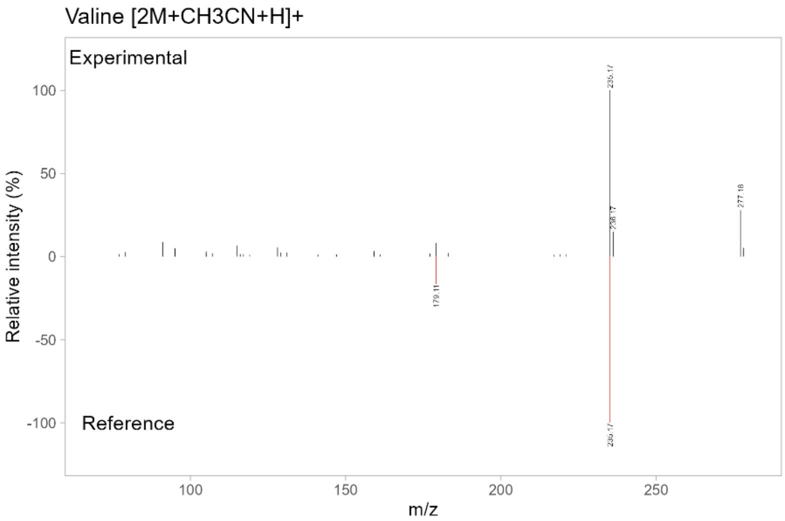

Supplementary Figure 23. MS/MS spectral match for valine against spectral library using MassBank of North America. Reference spectrum ID: splash10-000i-1190000000-77072ab21a5fad99c00c.

Supplementary results:

Other metabolite matches:

Cer 14:1;O2/10:0

Matched with mummichog using Lipids - Main Chemical Class RefMet pathway library

LPS 14:2

Matched with mummichog using Lipids - Main Chemical Class RefMet pathway library

Supplementary Table 1: OD_{600} values of bacterial isolates prior to metabolite extraction.

	**Replicate**		
**Isolate**	**A**	**B**	**C**
**KP001**	1.39	1.33	1.32
**EC002**	1.29	1.28	1.31
**KP003**	1.39	1.42	1.42
**EC004**	1.2	1.23	1.25
**EC005**	1.15	1.17	1.18
**KP006**	1.22	1.24	1.2
**KP007**	0.89	0.82	0.85
**KP008**	1.00	1.00	0.98
**KP009**	0.84	0.84	0.81
**KP010**	0.91	0.88	0.86
**EC011**	1.19	1.19	1.16
**KP012**	1.20	1.18	1.13
**EC013**	1.48	1.55	1.52
**KP014**	1.54	1.53	1.53
**EC015**	1.48	1.43	-
**EC016**	1.37	1.42	1.42
**KP017**	1.35	1.41	1.34
**EC018**	1.48	1.45	1.48
**KP019**	1.51	1.48	1.49
**EC020**	1.58	1.55	1.51
**EC021**	1.55	1.54	1.58
**KP022**	1.27	1.25	1.29
**KP023**	1.19	1.21	1.19
**EC024**	1.46	1.45	1.43
**KP025**	1.44	1.45	1.47
**EC026**	0.75	0.75	0.74
**KP029**	0.82	0.84	0.87
**KP030**	1.34	1.34	1.37
**KP031**	1.06	1.08	1.07
**EC032**	1.01	1.02	0.97
**EC033**	1.22	1.22	1.2
**KP034**	1.21	1.21	1.21

Supplementary Table 2: Selected XCMS preprocessing parameters optimised with IPO for endometabolome samples. Asterisked parameters were predefined and did not undergo optimisation.

**Processing step**	**Parameter**	**HILIC ESI-**	**HILIC ESI+**	**RP ESI-**	**RP ESI+**
**Peak picking**	ppm	9	8	12.504	9.25
**(centWave)**	peakwidth	13.4, 39	11.5, 39.2	11.5, 63	11.5, 60

	mzdiff	-0.00003728	-0.0131	0.0132164	0.0024672
	snthresh	10*	10*	10*	10*
	prefilter	3, 100*	3, 100*	3, 100*	3, 100*
	noise	1000*	1000*	1000*	1000*
**Retention time correction (obiwarp)**	profStep	1*	1*	1*	1*
	gapInit	0.6336	0.64	0.528	0.46
	gapExtend	1.9296	2.16	1.82	2.64
**Peak grouping (density)**	bw	9.08	17	0.25	17
	mzwid	0.00274	0.003	0.0126	0.003
	minfrac	0.25*	0.25*	0.25*	0.25*

Supplementary Table 3. Selected XCMS preprocessing parameters optimised with IPO for exometabolome samples. Asterisked parameters were predefined and did not undergo optimisation.

Processing step	Parameter	HILIC ESI-	HILIC ESI+	RP ESI-	RP ESI+
**Peak picking (centWave)**	ppm	10.1	7.5	9.9	8.48
	peakwidth	9.8, 63.5	9.8, 49	10.1808, 74	12, 48.5
	mzdiff	0.008128	-0.0043	0.001904	-0.00815
	snthresh	10*	10*	10*	10*
	prefilter	3, 100*	3, 100*	3, 100*	3, 100*
	noise	1000*	1000*	1000*	1000*
**Retention time correction (obiwarp)**	profStep	1*	1*	1*	1*
	gapInit	0	0.528	0.16	0.52
	gapExtend	2.64	2.04	2.64	2.25
**Peak grouping (density)**	bw	17	3.814	5.2	5.2
	mzwid	0.003	0.01066	0.003	0.003
	minfrac	0.25*	0.25*	0.25*	0.25*

Supplementary Table 4. Acquired resistance genes observed using whole genome sequencing.

ID	Species	MLST Type	Carbapenem	β-lactam	Aminoglycoside	Quinolone	Amphenicol	Phopshonic Acid	Tetracycline	MLS	Folate pathway antagonist
**KP001**	KP	ST273		blaOXA-1, blaTEM-1B, blaCTX-M-15, blaSHV-67	aph(6)-Id, aac(6')-Ib-cr, aph(3")-Ib	qnrB1, aac(6')-Ib-cr, OqxA, OqxB	OqxA, OqxB	fosA			sul2, dfrA14, OqxA, OqxB
**EC002**	EC	ST399		blaOXA-1, blaTEM-1B, blaCTX-M-15	aac(6')-Ib-cr, aadA1, aph(3")-Ib, aac(3)-IIa, aph(6)-Id	aac(6')-Ib-cr, qnrB1	catA1		tet(A)		sul2, dfrA14
**KP003**	KP	ST412		blaSHV-12		qnrS1, OqxA, OqxB	OqxA, OqxB	fosA			OqxA, OqxB
**EC004**	EC	ST648		blaCTX-M-15							
**EC005**	EC	ST131		blaCTX-M-15	aac(3)-IIa						
**KP006**	KP	ST234	blaKPC-2	blaOXA-9, blaSHV-1, blaTEM-1A	ant(2")-Ia, aac(6')-Ib, aadA1	OqxA, OqxB, aac(6')-Ib-cr	OqxA, OqxB, catB3	fosA			sul1, dfrA19, OqxA, OqxB
**KP007**	KP	ST234	blaKPC-2	blaSHV-5, blaOXA-9, blaTEM-1A	ant(2")-Ia, aac(6')-Ib, aadA1	OqxA, OqxB, aac(6')-Ib-cr	OqxA, OqxB, catB3	fosA			sul1, dfrA19, OqxA, OqxB
**KP008**	KP	ST258	blaKPC-2	blaSHV-182, blaTEM-1A, blaOXA-9	aadA1, aac(3)-IV, aac(6')-Ib, aadA2b, aph(4)-Ia, aph(3')-Ia	aac(6')-Ib-cr, OqxA, OqxB	catA1, cm1A1, OqxA, OqxB	fosA		mph(A)	sul1, sul3, dfrA12, OqxA, OqxB
**KP009**	KP	ST258	blaKPC-3	blaSHV-182, blaTEM-1A, blaOXA-9	aadA2, aph(3')-Ia, aadA2b	OqxA, OqxB	OqxA, OqxB, catA1	fosA		mph(A)	OqxA, OqxB, sul1, dfrA12
**KP010**	KP	ST258	blaKPC-2	blaSHV-182, blaTEM-1A, blaOXA-9	aac(6')-Ib-cr, aph(3')-Ia, aac(6')-Ib, aadA2	OqxA, OqxB, aac(6')-Ib-cr	OqxA, OqxB, catA1	fosA		mph(A)	OqxA, OqxB, sul1, dfrA12
**EC011**	EC	ST167		blaCTX-M-15, blaTEM-1B, blaOXA-1	aac(3)-IIa, aadA1, aac(6')-Ib-cr	aac(6')-Ib-cr	catA1		tet(A)		
**KP012**	KP	ST307		blaCTX-M-15, blaTEM-1B, blaOXA-1, blaSHV-28	aph(6)-Id, aac(6')-Ib-cr, aph(3")-Ib	qnrB1, aac(6')-Ib-cr, OqxA, OqxB	OqxA, OqxB	fosA	tet(A)	erm(B), mph(A)	OqxA, OqxB, sul2, dfrA14
**EC013**	EC	ST95									

**KP014**	KP	ST307		blaCTX-M-15, blaTEM-1B, blaOXA-1	aph(6)-Id, aac(6')-Ib-cr, aph(3'')-Ib	qnrB1, aac(6')-Ib-cr, OqxA, OqxB	OqxA, OqxB	fosA	tet(A)	erm(B), mph(A)	OqxA, OqxB, sul2, dfrA14
**EC015**	EC	ST410		blaCTX-M-15, blaOXA-1	aac(3)-IIa, aac(6')-Ib-cr	aac(6')-Ib-cr					
**EC016**	EC	ST101	blaNDM-1	blaOXA-2, blaTEM-1A	aac(6')-Ib-cr, armA, aph(6)-Id, aph(3')-VI, aadA2, aac(3)-IIa, aph(3'')-Ib	aac(6')-Ib-cr	catA1		tet(D)	msr(E), mph(E)	sul1, dfrA29, dfrA12
**KP017**	KP	ST14	blaNDM-1	blaOXA-1, blaOXA-9, blaTEM-1A, blaCTX-M-15	aadA1, aac(6')-Ib, aph(3'')-Ib, aac(3)-IId, aac(3)-IIa, aac(6')-Ib-cr, aph(6)-Id	aac(6')-Ib-cr, OqxA, OqxB	catA1, cm1A1, OqxA, OqxB	fosA		ere(A)	sul1, sul2, dfrA1, OqxA, OqxB
**EC018**	EC	ST410	blaOXA-48	blaCMY-146					tet(B)	erm(B), mph(A)	dfrA14
**KP019**	KP	ST353	blaOXA-48	blaTEM1-B, blaSHV-*	aph(3')-Ia	OqxA, OqxB	OqxA, OqxB, catA1	fosA	tet(C)	ere(A)	OqxA, OqxB, dfrA14, dfrA5, sul1
**EC020**	EC	ST2083	blaOXA-181	blaTEM-1B	aph(3'')-Ib, aph(6)-Id, aac(3)-IId	qnrS1			tet(B)		
**EC021**	EC	ST2083	blaOXA-181	blaTEM-1B, blaCMY-42	aph(3'')-Ib, aph(6)-Id, aac(3)-IId	qnrS1			tet(B)		sul2
**KP022**	KP	ST14	blaNDM-1, blaOXA-232	blaTEM-1A, blaOXA-1, blaCTX-M-15	armA, aadA2, aph(3')-VI, aac(6')-Ib-cr	OqxA, OqxB, aac(6')-Ib-cr	OqxA, OqxB	fosA		msr(E), mph(E)	OqxA, OqxB, sul1, dfrA12, dfrA14, dfrA1
**KP023**	KP	ST14	blaNDM-1, blaOXA-232	blaTEM-1A, blaOXA-1, blaCTX-M-15	armA, aadA2, aph(3')-VI, aac(6')-Ib-cr	OqxA, OqxB, aac(6')-Ib-cr	OqxA, OqxB	fosA		msr(E), mph(E)	OqxA, OqxB, sul1, dfrA12, dfrA14, dfrA1
**EC024**	EC	ST73									
**KP025**	KP	ST82						fosA			
**EC026**	EC	ST93									
**KP029**	KP	ST3		blaSHV-*		OqxA, OqxB	OqxA, OqxB	fosA			OqxA, OqxB
**KP030**	KP	ST20	blaNDM-5	blaTEM1-B, blaSHV-187	rmt(B), aadA2	OqxA, OqxB	OqxA, OqxB	fosA		erm(B)	OqxA, OqxB, sul1, dfrA12

**KP031**	KP	ST14	blaNDM-1	blaCTX-M-15, blaOXA-1, blaSHV-*, blaTEM-1A, blaOXA-9, blaCMY-4	aadA1, aph(6)-Id, aac(3)-IIa, aac(6')-Il, aac(3)-Iid, armA, aph(3")-Ib, aac(6')-Ib-cr	aac(6')-Ib-cr, OqxA, OqxB	catA1, cm1A1	fosA		msr(E), ere(A), mph(E)	OqxA, OqxB, sul1, sul2, dfrA1
**EC032**	EC	ST205	blaNDM-7	blaOXA-1, blaCTX-M-15	aac(6')-Ib-cr, aadA2, aadA1	aac(6')-Ib-cr				mph(A)	suk1, dfrA1, dfrA12
**EC033**	EC	ST361	blaNDM-5	blaCMY-42	aadA2				tet(A)	mph(A)	sul1, dfrA12
**KP034**	KP	ST353	blaOXA-48	blaTEM-1B, bla-SHV*	aph(3')-Ia	OqxA, OqxB	catA1	fosA	tet(C)	ere(A)	dfrA14, dfrA5, sul1

KP = *K. pneumoniae*, EC = *E. coli*, MLS = Macrolide, Lincosamide and Streptogramin B

Supplementary Table 5. Mass spectral features detected in endo- and exometabolome samples across each ion and chromatographic mode and features remaining after feature selection.

	Endometabolome				Exometabolome			
**Analytical method**	HILIC (ESI+)	HILIC (ESI-)	RP (ESI+)	RP (ESI-)	HILIC (ESI+)	HILIC (ESI-)	RP (ESI+)	RP (ESI-)
**Detected features**	21,728	14,779	18,678	4,510	22,513	15,948	31,105	19,014
**Detected features after quality filtering (and blank subtraction for exometabolome samples)**	8,696	9,834	6,564	2,143	340	460	159	220
**Selected features**	16	42	13	0	36	2	14	10
